# Initial programme theory for community-based ART delivery for key populations in Benue State, Nigeria: a realist evaluation study

**DOI:** 10.1186/s12889-023-15774-w

**Published:** 2023-05-12

**Authors:** Olujuwon Ibiloye, Tom Decroo, Josefien van Olmen, Caroline Masquillier, Prosper Okonkwo, Lutgarde Lynen, Plang Jwanle, Sara Van Belle

**Affiliations:** 1 Institute of Tropical Medicine, Antwerp, Belgium; 2 APIN Public Health Initiatives, Abuja, Nigeria; 3 Belgian Research Foundation Flanders, 1000 Brussels, Belgium; 4 University of Antwerp, Antwerp, Belgium

**Keywords:** Key populations, HIV, Community-based antiretroviral therapy, ART, Realist evaluation, Programme theory

## Abstract

**Background:**

The community-based antiretroviral therapy delivery (CBART) model was implemented in Benue State in Nigeria to increase access of key populations living with HIV (KPLHIV) to antiretroviral treatment. Key populations (KP) are female sex workers, men who have sex with men, persons who inject drugs, and transgender people. Evidence shows that the CBART model for KP (KP-CBART) can improve HIV outcomes along the cascade of HIV care and treatment in sub-Saharan Africa. However, how KP-CBART works, for whom, why, and under what circumstances it generates specific outcomes are not yet clear. Therefore, the aim of this study is to identify the initial programme theory (IPT) of the KP-CBART in Benue State using a realist approach.

**Method:**

The study design is exploratory and qualitative, exploring the implementation of KP-CBART. We reviewed the intervention logic framework & guidelines for the KP-CBART in Nigeria, conducted a desk review of KP-CBART in Sub-Saharan Africa (SSA) and interviewed programme managers in the Benue HIV programme between November 2021 and April 2022. Findings were synthesized using the Context-Mechanism-Outcome (CMO) heuristic tool to explain the relationship between the different types of CBART models, contextual factors, actors, mechanisms and outcomes. Using a generative causality logic (retroduction and abduction), we developed, following a realist approach, CMO configurations (CMOc), summarized as an empirically testable IPT.

**Result:**

We developed 7 CMOc and an IPT of the KP-CBART. Where KPLHIV receive ART in a safe place while living in a setting of punitive laws, harassment, stigma and discrimination, KP will adhere to treatment and be retained in care because they feel safe and trust the healthcare providers. Where KPLHIV are involved in the design, planning and implementation of HIV services; medication adherence and retention in care will improve because KP clients perceive HIV services to be KP-friendly and participate in KP-CBART.

**Conclusion:**

Implementation of CBART model where KPLHIV feel safe, trust healthcare providers, and participate in HIV service delivery can improve medication adherence and retention in care. This programme hypothesis will be tested and refined in the next phase of the realist evaluation of KP-CBART.

**Supplementary Information:**

The online version contains supplementary material available at 10.1186/s12889-023-15774-w.

## Background

Nigeria has the fourth highest number of people living with HIV/AIDS in the world with an estimated 1.7 million (1.3 M – 2.3 M) people living with HIV (PLHIV) [1] and 86,000 new infections in 2020 [2, 3]. According to the Nigeria National HIV/AIDS Indicator Survey, the national HIV prevalence is 1.3% (males- 1%; females – 1.6%) for those within age 15–49 years in 2019 [2]. However, HIV is more prevalent among members of key populations (KP). KP are groups of individuals who are at the highest risk of contracting and transmitting HIV, irrespective of the epidemic type or local context [4].

KP generally include female sex workers (FSW), men who have sex with men (MSM), persons who inject drugs (PWID) and transgender people (TG). The 2020 Integrated Behavioural and Biological Surveillance Surveys (IBBSS) estimated that the national HIV prevalence rate among FSW, MSM, TG, and PWID was 16.7%, 25%, 28.8% and 10.9% respectively [5]. FSW and transgender women have 30 and 14 times more risk of acquiring HIV compared to adult women. MSM have 28 times higher risk compared to adult men while PWID have a 35 times higher risk than adults who do not inject drugs [6]. In 2020, KP and their sexual partners accounted for 65% of new HIV infections in the world and 39% in Sub-Saharan Africa (SSA) [7]. In Nigeria, KP individuals make up only 3.4% of the country population, yet accounted for about 32% of new HIV infections in 2014 [8]. These findings were also shown in previous IBBSS (in 2007; 2010, 2014, and 2020). The 2020 IBBSS emphasised the importance of KP as drivers of the HIV epidemic and the need to intensify efforts to increase HIV testing and treatment coverage.

In Nigeria, KP are generally underserved and have poor access to effective ART. ART coverage among KP is still low when compared to the general population in the country. In Nigeria, in 2021, ART coverage among adults and children was 90% while while ART coverage for FSW, PWID, TG, and MSM was 23.7%, 25%, 19.5%, and 26.3% respectively [6]. Poor access to effective ART can be explained by social and legal barriers, stigma and discrimination, criminalisation and violence confronting KP individuals [4, 9, 10]. Therefore, to improve access of KPLHIV to ART, interventions must be tailored to reduce or eliminate specific barriers to treatment and to meet individual health needs of KP. The World Health Organization (WHO) recommended differentiated ART service delivery (DSD) to optimise care for KPLHIV on ART [4, 11]. DSD models such as CBART models, peer-led and health care worker (HCW)-led models were recommended to improve access to care and treatment outcomes among KPLHIV.

### Rationale

Most of the evidence on CBART models is from studies targeting the general population. There is still inadequate evidence on the effectiveness of the CBART models of service delivery for KP (KP-CBART) in SSA, including Nigeria. Two previous reviews described the effects of KP-CBART along the cascade of HIV care and treatment in Sub-Saharan Africa. The effects of KP-CBART in Tanzania, Zimbabwe, Benin, Nigeria, and Congo were described [12, 13]. One of the studies, Atuhaire et al., a systematic review, focussed on the FSW and the other, Ibiloye et al., (scoping review) on MSM, FSW, PWID, and TG [12, 13]. These studies showed that treatment outcomes in KP-CBART were as good as the facility-based care [12, 13]. However, in studies on the effects of KP-CBART, there is no evidence on how, why, and for whom the model worked, and in what contexts it worked. The facilitators and barriers of KP-CBART are yet to be explored in depth. Therefore, we sought to evaluate the KP-CBART model in Benue in Nigeria, using the realist evaluation method [14] to generate evidence on the adaptation and scale-up of the models to meet the health needs of KP for optimal impact in Benue Nigeria and in similar settings in SSA. The research question is “how, why, for whom, and in what context conditions do community-based ART models of service delivery contribute to observed clinical outcomes among key populations in Benue State, Nigeria?”.

### Realist evaluation method

We adopted the realist evaluation approach for our study. The realist evaluations are theory-based evaluation developed by Pawson and Tilley in 1997, it is grounded in the paradigm of scientific realism. The realist evaluations approach is based on’’ the assumptions that programme or policy interventions work under certain conditions and are influenced by the way different stakeholders respond to them’’ [15]. This approach follows a generative causality model and suitable for complex interventions to assess how and why interventions contribute to outcomes in different contexts. Therefore, the realist evaluation approach seeks to answer the following questions: what works or does not work?’, ‘for whom (and to what extent?’, ‘in which circumstances does it work?’, ‘how and why does it work?’. What is specific to the realist evaluations approach is the analysis through a configurational heuristic context-mechanism-outcome configuration. It develops a contextual that aims to explain the mechanisms triggered that generate different outcomes.

We aim to conduct a realist evaluation of the KP-CBART programme and It will be conducted in 4 phases. The first phase of the realist evaluation is the development of the initial programme theory (IPT) about KP-CBART programme in Benue state from sources such as existing theories, previous studies, documentary analysis, etc. Also, potential CMO configurations are developed and testable hypotheses are generated during this phase. In the second phase, we will use appropriate methods (i.e. qualitative mixed methods) to collect data on the hypothesized CMO configurations. During the third phase (data analysis and hypothesis testing), data are collected and outcome patterns are used to examine the hypothesised CMO configurations. In the fourth phase, the proposed CMO configurations will be refined. This refinement is based on findings from previous phases: patterns will be analysed and propositions will be examined and refined. The realist research cycle of this study is fully described in the published protocol (to insert reference when published in December).

### Aim

In this paper, we report on the first phase of the realist evaluation of the KP-CBART in Benue State, Nigeria. We present the initial programme theory (IPT) of the KP-CBART explaining how, why and in which context conditions the KP-CBART model contributes to improved clinical outcomes for key populations. Our primary question was: what are the mechanisms and context conditions that drive succesful community-based ART implementation and how do these lead to better retention in care, treatment adherence, and viral suppression amongst which categories of KP?

### Research methods

The report of this study was guided by the Realist And Meta-narrative Evidence Syntheses: Evolving Standards (RAMESES) II) for realist evaluations [16]. The checklist contains 20 items that should be included in reporting standards for realist evaluation [16]. Although this paper did not report the items in the order or sequence as in the checklist, all the items were used.

### General or study setting

Benue state is located in northcentral region of Nigeria and the state capital is Makurdi. It has an area of 34,059 km2 (13,059 sq mi) and an estimated population of 4,253,641 in 2006 [17]. The State consists of 23 Local Government Areas and there are two main ethnic groups, namely the Idoma and the Tiv people. Our Study sites are situated in Makurdi, Gboko, Otukpo and Gwer West LGAs in the state [18].

In collaboration with donor agencies (i.e. PEPFAR CDC) and implementing partners, the State Agency for the control of AIDS and the State Ministry of Health provide ART services to KPLHIV through the various community-based ART delivery models and KP-friendly public health institutions in the state. Despite increasing access to ART in the state, KP individuals continued to experience poor access to HIV prevention, care, and treatment services.

### The community-based ART model for key population in Benue State, Nigeria

In 2016, a community-based ART service delivery model for key populations (KP-CBART) was established in Benue State, Nigeria. This type of differentiated ART service delivery (DSD) is also known as the Community-Based One Stop Shop Clinic Model in Nigeria [19]. The programme is part of the national HIV programme that is being implemented by partners, the National Agency for the Control of AIDS and the Federal Ministry of Health with support from PEPFAR through the United States Centres for Disease Control and Prevention.

The KP-CBART in Benue State provides ART through three different modalities. Patients either receive ART through the One Stop Shop clinic (OSS) or community Drop-In Centers (DIC) or both OSS and community Outreach Venues (OV).

**Fig. 1:**
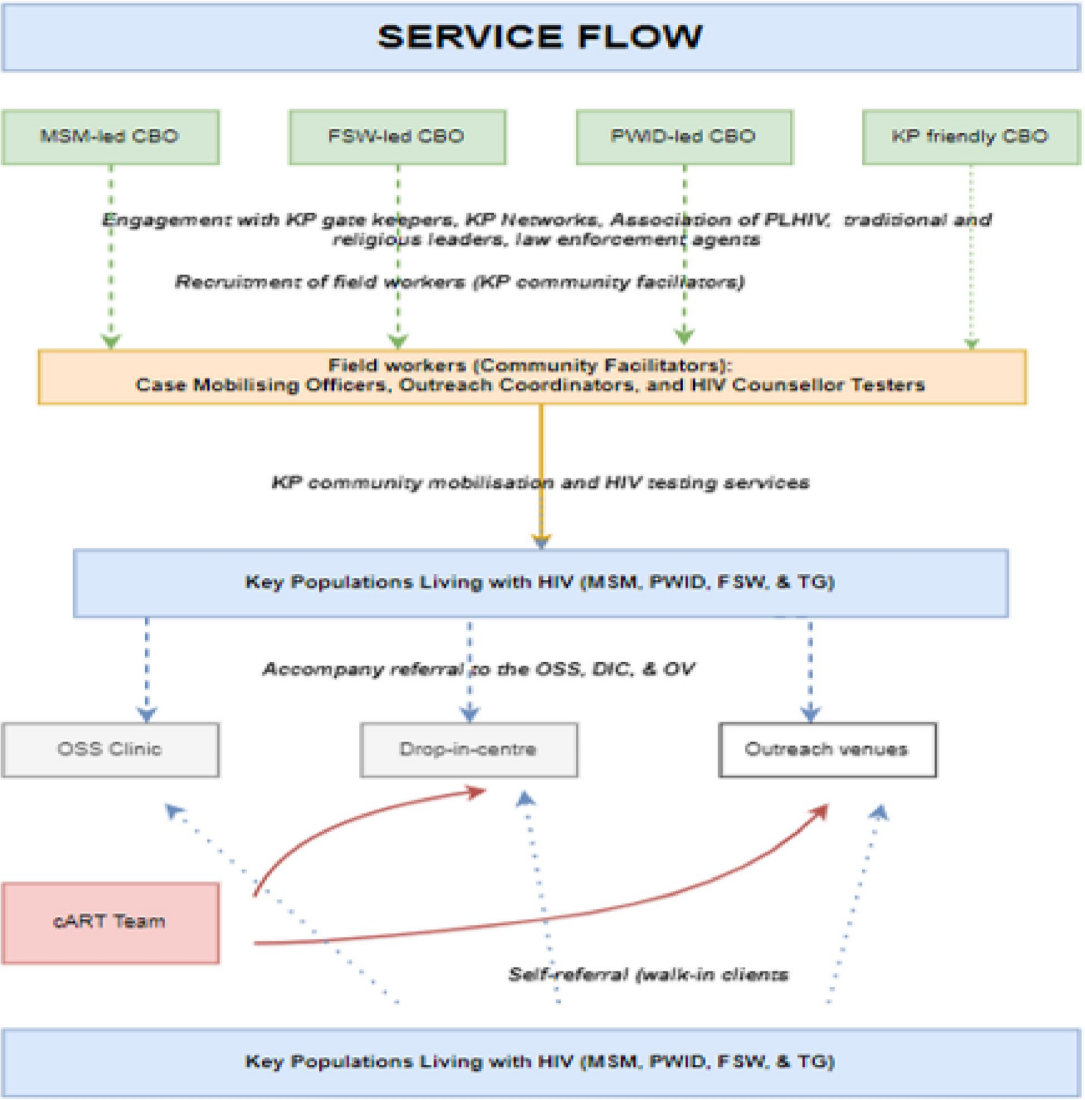
Description of the Community-based ART delivery models for key populations in Benue Nigeria, KP-key population,, cART- community/mobile ART, CBO-community-based organization,MSM-men who have sex with men, female sex workers, TG-transgender, PWID – persons who inject drug

Figure 1 and Table 1 below depicts the model as initially designed. The OSS clinics are primary health care structures that provide comprehensive HIV services strictly to KP in an environment free of stigma and discrimination [19]. OSS are operated by community members and community-based organizations (CBO). The OSS provide HIV prevention, care, and treatment support and protection services for KP. Professional health workers (i.e. medical officer, pharmacist, nurse, medical laboratory scientist, and adherence counselor) are employed to provide HIV services in the OSS. The DIC is a mini-OSS and is led by community healthcare providers. The OV are hotspots or agreed locations within the community where ART is provided to KP. The outreach venue is served by a mobile multidisciplinary health team to ensure ART [19].

**Table 1:** Description of community-based ART models for key populations in Benue State, Nigeria

	**Community drop-in-centre (DIC)**	**Community outreach venues with mobile ART team**	**Community-based one stop shop clinic**
Target population	FSW, MSM, PWID	FSW, MSM, PWID	FSW, MSM, PWID
HIV care delivery point	A safe place where KP can meet/gather for social and clinical activities	DIC plus mobile health team (clinician, nurses and peer educators) to homes, and hotspots including hotels, brothels, bunkers	Provision of KP friendly health care services in a trusted community ART centre
Location	Semi-urban	Rural or semi-urban	Urban
Operation hours	Daily	Once or twice per week	5-days per week
Package of services	Peer-led HIV counselling and testing, antiretroviral treatment, accompany referral, tracking of clinic defaulters by peers and network, provision of condoms, KP sensitization training for HCWs	Peer-led HIV counselling and testing, antiretroviral treatment, accompany referral, tracking of defaulters by peers and network, provision of condoms, KP sensitization training for HCWs	Peer-led HIV counselling and testing, antiretroviral treatment, accompany referral, tracking of clinic defaulters by peers and network, provision of condoms, KP sensitization training for HCWs, cervical cancer screening)
Care providers	**Health professionals:** community ART Nurse **Community/Lay health workers:** Peer educators, community mobilising officers, adherence counsellors	**Mobile ART Team (mART):** ART Clinician, Pharmacist, and Medical Laboratory Scientist from the OSS clinic **Community/Lay health workers:** Peer educators/community mobilising officers	**Health professionals:** ART Clinician, ART Nurse, Pharmacist and Medical Laboratory Scientist **Community/Lay health workers:** Peer educators, community mobilising officers, adherence counsellors
Roles of KP community or lay HCWs in HIV care	Community sensitization and mobilisation, HTS, adherence counselling, ART refill and referral	Community sensitization and mobilisation, HTS, adherence counselling, ART refill and referral	Community sensitization and mobilisation, HTS, adherence counselling, ART refill and referral

OSS clinics are established in areas with an estimated large number of KP, such as in the capital of local government areas, usually an urban setting. DICs are established in semi-urban areas. DICs are present in locations or towns without OSS. Medical records of KP clients in the community outreach venues is domiciled at the OSS, meaning they can access ART services through the OSS and/or community outreach venues.

In the KP-CBART, health care professionals work with lay healthcare workers (lay HCW) attached to a network of KP-led and KP-competent civil society organizations. Mobile ART teams (comprised of a medical doctor, pharmacist, and a medical lab scientist) collaborate with peer navigators and outreach coordinators to conduct ART outreaches and provide HIV care to KPLHIV in drop-in-centres (usually offices of community-based organizations and primary health centres) and at outreach venues or hotspots for members of KP (hotels, club houses, and etc.) (APIN Public Health Initiatives: Technical proposal and framework for key populations, unpublished).

The KP-CBART is designed to be implemented mainly by KP-led CBOs whose board members and staffs are primarily comprised of KP and professional healthcare workers. Donors supported partners to strengthen institutional capacity of KP-led and KP-competent CBOs for effective programming (financial and management systems) to build long term self-efficacy and to deliver HIV services such as HIV case finding, enrolment on ART and retention in treatment, mitigation of stigma and discrimination, and syndromic STI management.

KP-led CBOs engage staff members who have a deep understanding of the geographic, social and cultural factors affecting the programme beneficiaries (KP clients). KP individuals as peer outreach workers and peer navigators provide case finding and case management. These KP individuals offer HTS, and refer HIV diagnosed clients to the OSS or DIC for ART enrolment (APIN Public Health Initiatives: Technical proposal and framework for key populations, unpublished). They are also responsible for retention on ART for already enrolled KP, by organizing support group meetings and sending appointment reminders (phone SMS and/or phone call and home visit) to clients for ART refill and VL testing (APIN Public Health Initiatives: Technical proposal and framework for key populations, unpublished). They also mobilise patients during ART outreaches (APIN Public Health Initiatives: Technical proposal and framework for key populations, unpublished).

### Study design

The exploratory qualitative study design was adopted to describe KP-CBART in Benue State Nigeria and to develop the IPT [20]. A programme theory refers to an abstracted description or diagram that lays out what a programme (or family of programmes or intervention) comprises and how it is expected to work [21]. Central to the elicitation of the programme theory is the development of the Context-Mechanism-Outcome configuration (CMOc) and the IPT for the KP-CBART.

The original programme theory developed by the programme was not realist in nature and need to be developed and refined so that it becomes a realist programme theory (that is, addressing all of context, mechanism and outcome) [21]**.** The IPT identifies a set of explicit or implicit assumptions by stakeholders about how and why the intervention might work and be implemented through literature review, conversations/interviews, and documents [14, 16]. The IPT will be tested and refined throughout the 2^nd^, 3^d^, and-4^th^ phase of the realist evaluation.

The first phase of the realist study is the development of the IPT for the KP-CBART models [22]. Realist evaluation and research is a theory-driven research methodology that will make the programme theory of the KP-CBART explicit by describing and testing the IPT on how, and for whom, the model works (or does not work) and under what context conditions (context), it is expected to work [14, 23].

**Fig. 2:**
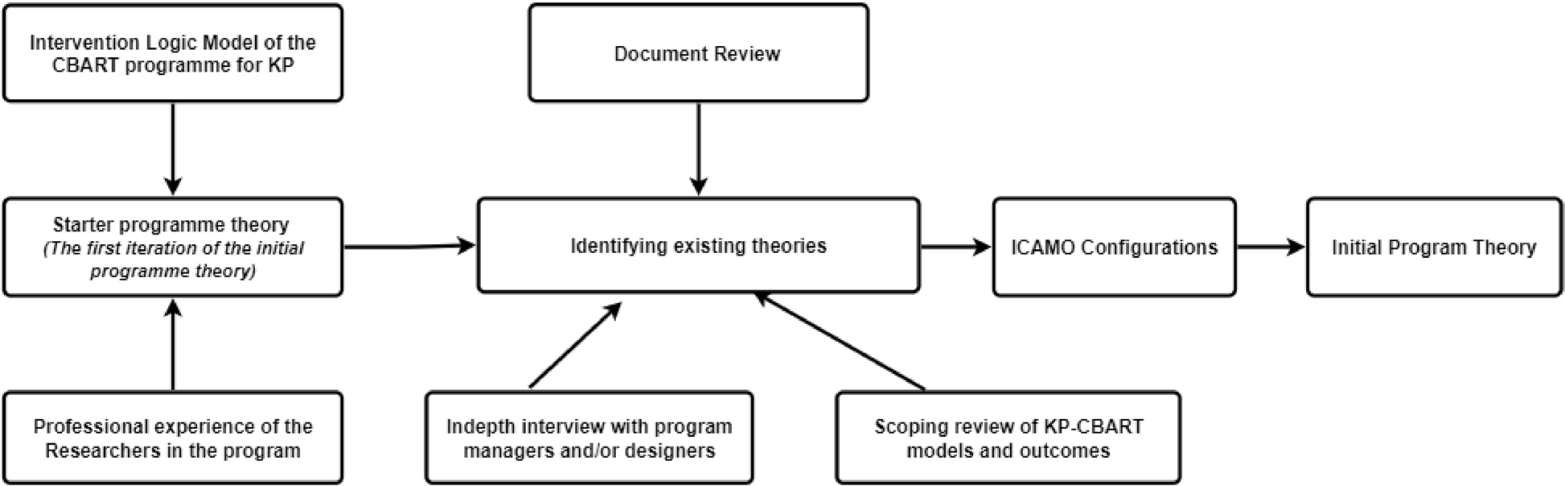
Steps taken to elicit the Initial Programme Theory for the community-based HIV service delivery model for KP in Benue state, Nigeria

We followed the steps of developing an IPT as set out by Mukumbang et al. [22]. Figure 2 and Table 2 below summarize the various activities that were conducted to develop the IPT and their objectives.

**Table 2:** Summary of activities to develop the Initial Programme Theory of the KP-CBART model

	**Activity**	**Aim**
Theory gleaning phase (Data collection)	**Step 1:** Review of the intervention logic model and professional experience of the researcher	•To elicit the starter programme theory (the pre-existing theory of KP-CBART informed by the intervention logic model) •To explain how different programme activities will contribute to a series of results that produce the intended outputs and outcomes
	**Step 2:** Literature review of policy and programme documents on the implementation policy, guidelines, framework and journal articles on community-based HIV prevention, care and treatment for key populations	•To describe the history and evolution of KP-CBART model implementation in in SSA including Nigeria and the policy environment •To describe the programme strategies and activities and to identify contextual factors
	**Step 3:** Scoping review on outcomes along the cascade of HIV care and treatment continuum	•To identify the outcomes of the CBART intervention for KP
	**Step 4:** In-depth interviews with programme managers	•To elicit the starter programme theory programme managers’ assumptions (folk theories) •To map out programme- specific CMO configurations •To refine the starter programme theory
	**Step 5**: Review of an existing programme theory for antiretroviral treatment adherence club programme in South Africa to inform the CMOc and programme theory for KP-CBART	To adapt the existing programme theory of the ART adherence club intervention to suit the KP-CBART programme Nigeria, including the mechanisms
Eliciting the initial programme theory (Data analysis)	**Step 6:** Synthesis of results	Triangulation of data and analysis of Context-Mechanism-Outcome configurations for KP-CBART programme and the IPT

*KP-CBART* Community based ART model for key population, *IPT* Initial programme theory, *ART* Antiretroviral therapy, *CMOc* Context-mechanism-outcome configurations, *KP* Key populations

### Step 1. Development of the starter programme theory

**Fig. 3:**
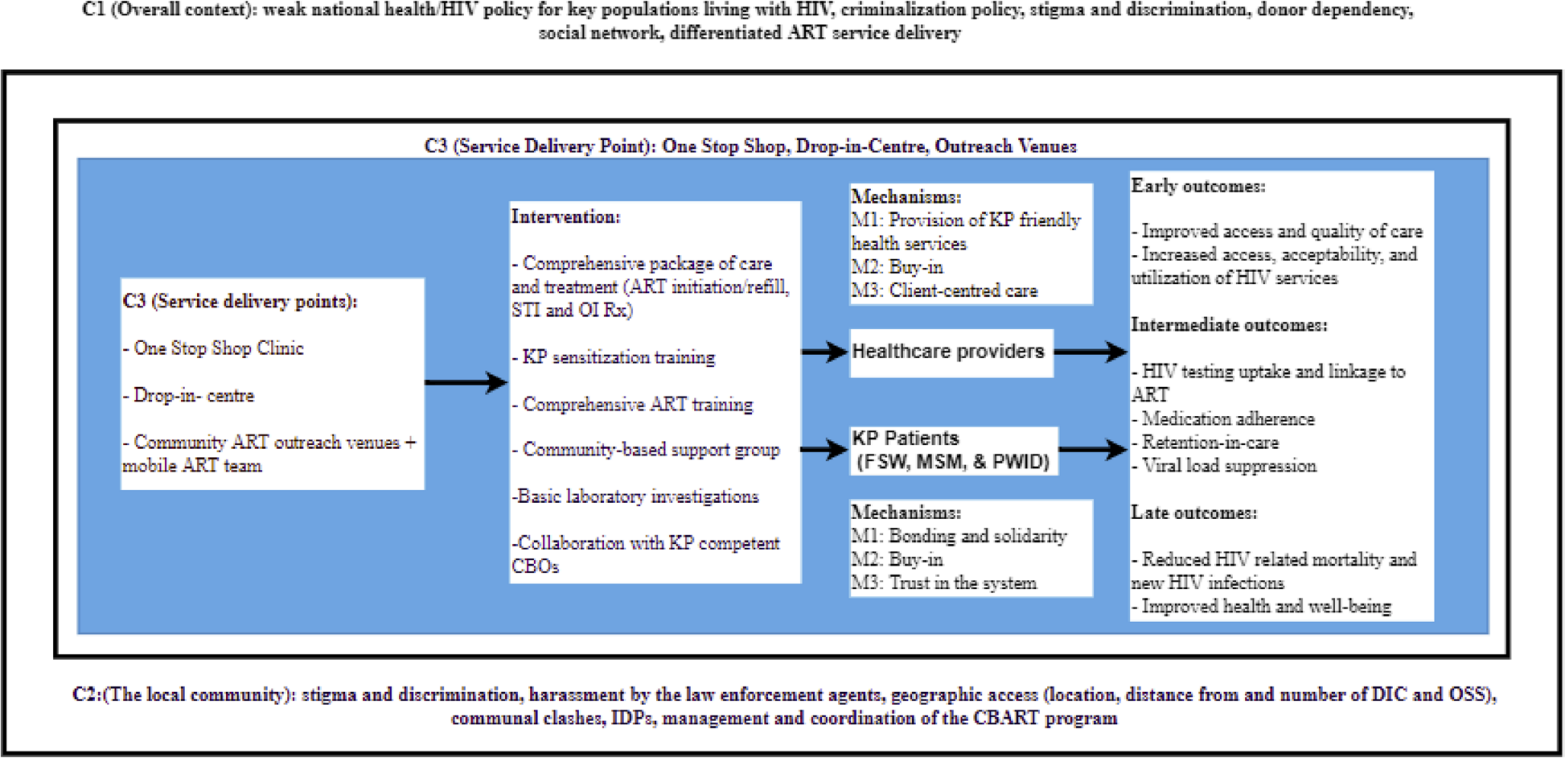
Starter programme theory for the CBART model for KP developed during protocol development, CBART-community-based antiretroviral therapy, KP-key population, KP-CBART- community-based ART service delivery models for KP, C-context, mART- mobile ART, CBO-community-based organization, M- mechanism, Rx-treatment

Figure 3 presents the starter programme theory for the CBART model for KP developed during the first step of the study. The starter programme theory is the pre-existing programme theory before the realist evaluation and it is informed by the intervention logic model or framework. The starter programme theory (Fig. 3) explains the assumptions regarding how the CBART model would achieve better health outcomes for key population groups (KP) compared to the facility-based ART.

Our starter programme theory was informed by the professional experience of the principal researcher and lead author as an HIV programme officer in the programme, the review of the intervention logic model developed by the programme designers, and the activities workplan. The researcher and lead author was employed by the implementing partner organization, APIN Public Health Initiatives. He provided technical support to the community-based organizations, and mentoring and coaching of the healthcare workers working in the KP-CBART programme.

This phase of theory development aimed at understanding the context of programme implementation: (1) individual clients, (2) service delivey points and the health facility, (3) local community health system, and the different intervention components such as: the model of ART service delivery or the actual point of service delivery (One Stop Shop (OSS), community Drop in centre (DIC), and Outreach Venues (OV), interventions (HIV testing and treatment services, peer support group and peer education, KP sensitization training, ART trainings, basic laboratory investigation), key actors (KPLHIV and healthcare providers), causal mechanisms (e.g. buy-in, trust in the system) and outcomes (e.g. linkage to ART, medication adherence).

To develop the starter programme theory and better understand the programme intervention logic, we used a series of questions recommended by the guidance produced by the RAMESES project (funded by the United Kingdom's National Institute of Health Research's Health Services and Delivery Research) [15] to map out the interventions, contexts, actors, causal mechanisms, and the outcomes (supplementary Table 1).

The starter programme theory of the CBART programme can be formulated as follows:

1. In resource-constrained settings with an unfavorable policy environment (in terms of criminalization policy against KP activities), potential arrest by police, poor geographic access, inadequate number of KP friendly healthcare facilities within the state/community and low levels of trust between the health workers and members of KP (C)**,** decentralisation of ART service delivery to KP communities together with training of HCW on KP sensitization and comprehensive ART will enhance trust (M) and psychological safety (M) in the programme and encourage (M) KP to access HIV care and treatment services and this will improve uptake and utilisation of these services and retention-in-care (intermediary outcome) (Fig. 3). Optimal HIV prevention and treatment for KP will translate to better health outcomes and well-being for KP (final outcome).
2. Involvement of KP community and lay workers in all components (e.g. accompany referral for ART, HIV testing and linkage to ART, medication adherence, ART refill, clients tracing, and etc.) of a comprehensive HIV care package (C) would make HIV service KP-friendly (M) and thus, improve long term outcomes/sustained engagement of KPLHIV in care and clinical outcomes (O).

### Step 2: Desk review of operational guidelines and frameworks on HIV care

We conducted a desk review of programme documents (the implementation policy and guidelines, progress report) and peer-reviewed articles on community-based HIV prevention, care, and treatment for key populations in SSA. We searched and extracted articles between November 2021 and January 2022. Documents that described the community-based ART programme for KP in sub-Saharan Africa were obtained from different sources with the objective of describing the KP-CBART programme, the objectives, key strategies and activities. We analysed programme documents (project implementation plan, progress report, and the Notice of Funding Opportunity (NOFO) and searched PubMed, Google scholar, Google search, and Web of Science for articles on KP-CBART using the terms “key populations”, “community based ART”, “HIV”, and “Africa”. We also specifically searched the website of the KP implementing organizations in Nigeria (Society for Family Health, Population Council, APIN Public Health Initiatives, Heartland Alliance, and FHI 360). The prisma diagram of the literature search and study selection process is as shown in supplementary Fig. 1.

Web search yielded international guidelines for HIV prevention, care, and treatment for KP, policy documents, national implementation guidelines for MSM, FSW, and PWID, and journal articles that described the KP-CBART intervention and its effect on clinical outcomes. The supplementary Table 2 described the characteristics of papers included in the desk-review. Information obtained from this review helped to identify and explain the interaction between the various contextual elements, actors, mechanisms, and outcomes that are at play within the programme and also, guide the development of the semi-structured in-depth-interview guide used to interview the programme managers and/or programme designers.

Document analysis was guided by the CMO heuristic tool, to obtain information on the relevant contextual factors, mechanisms, and outcomes [24].

### Step 3: Scoping review of KP-CBART on outcomes

We previously published a scoping review on the outcomes of KP-CBART along the cascade of HIV care and treatment in sub-Saharan Africa, to identify some of the outcomes and context conditions of the CBART intervention for KP [13]. The purpose of the scoping review was to summarize the evidence on the effect of CBART along the continuum of HIV care among KP in sub-Saharan Africa. Each paper in the study (scoping review) was re-analysed to identify the contextual factors and the causal mechanisms for the observed outcomes, using the CMO heuristic framework.

### Step 4: In-depth interviews with programme managers and implementers

We conducted 13 in-depth interviews with programme managers and designers to elicit their assumptions regarding contextual factors and the causal mechanisms that influence the programme outcomes. The first author (OI) and an assistant conducted in-depth interviews with programme managers between December 2021 and January 2022. Interviews were scheduled and held in the offices of the programme designers and managers. Thirteen programme managers and designers were purposively selected for a face-to-face interview using a semi-structured interview guide (Supplementary Table 3)*.* The interview guide was piloted with 3 programme managers, following which the interview guide was adapted based on verbal feedback from study respondents and findings from data analysis. Programme designers and managers working with KP-led CBOs, partner organizations, and government agencies were interviewed to understand their views and perspectives about the KP-CBART programme and the overall programme objectives, and how the programme intends to achieve its objectives. Barriers and facilitators to achieving programme objectives were also explored. Following the transcription and coding of the transcripts, follow-up calls was made to some of the interviewees to confirm their assumptions.

The Table 3 below shows the list of programme designers and managers that were interviewed:

**Table 3:** Programme Designers or Managers interviewed and their organizations (Donor, Implementing partner, KP-led Community based organizations, and government agencies)

Type of Organization	Number of organization	Number of Project Managers	Description of Project Managers
Implementing partner	2	8	KP Thematic Leads (Clinicals, Community and Prevention Programme Specialist)
Community-based Organization (FSW focused)	1	1	Community KP Programme Manager
Community-based Organization (MSM focused)	1	1	Community KP Programme Manager
Community-based Organization (PWID focused)	1	1	Community KP Programme Manager
Community-based Organization (focus on all KPs)	1	1	Community KP Programme Manager
Benue State Agency for STI and AIDS Control Programme (SASCP)	1	1	HIV Programme Manager
**Total number of interviewees**		**13**	

### Step 5: Identification and revision of an existing programme theory to suit the KP-CBART programme in Benue, Nigeria.

Between 2015 and 2018, Mukumbang et al. [25] conducted a realist evaluation of the antiretroviral treatment adherence club intervention in the Western Cape Province, South Africa. This ART adherence club intervention shared many attributes with the KP-CBART programme in Benue State, Nigeria. Table 4 below describes the similarities and differences between the two models of care. The two HIV programmes have the same programme objectives to increase access of PLHIV to ART and to ensure medication adherence and retention in HIV care. Mukumbang and co-authors explained the IPT and the refined programme theory of the adherence club modalities in Western Cape in their publications [25, 26]. The IPT of the ART adherence club was developed based on “an exploratory qualitative study of programme designers and managers’ folk theories [14], document review of the design, a systematic review of group-based ART adherence models in SSA [27]*,* and a scoping review of the social, relational, cognitive, and behavioural theories that explained adherence to ART” [25].

**Table 4:** Similarities and differences between the KP-CBART model in Benue State, Nigeria and ART adherence club model in Cape Town, South africa

	**KP-CBART model (2016)**	**ART adherence club model (2010)**
Location of service delivery	Benue, Nigeria	Western Cape Province, South Africa
Target population	Key populations living with HIV (mention categories)	Stable patients older than 18 years or more
Policy environment	•Criminalization and punitive regulation of sex work •Criminalization of homosexual activities (imprisonment up to 14 years) •Hostile religious environment	•Criminalization and punitive regulation of sex work •Laws penalising same-sex sexual acts decriminalized, or never existed
Health system	Vertical HIV programmes delivered by NGOs, not yet integrated into the public health system	The HIV programme is integrated into the public health system and is delivered by both the government and the NGOs
Service delivery context	A safe space free of stigma and discriminination	A conducive, user-friendly environment to sensitize group members on health and wellbeing
Type of differentiated service delivery model	Community-based, the same state but different locations	Facility-based; specific fixed location
Goal	To improve access of KP to quality HIV services	To streamline treatment and care of stable patients
Objectives	To address poor retention in care and sub-optimal adherence to ART among KP	To address poor retention in care, sub-optimal adherence to ART, and health care facility congestion among stable patients
Implementation phase	Scale-up	Scale-up (roll out)
Health care providers	A mix of lay health workers and professional health workers	Health worker-led
Support group meetings and peer support	Yes	Yes
Collaboration with CBOs	Collaboration with KP-led CBOS	Collaboration with local patient-led CBOs

*CBART* community-based antiretroviral therapy, *KP* Key population, *KP-CBART* Community-based ART service delivery models for KP, *CBO* Community-based organization, *ART* Antiretroviral therapy

Therefore, we decided to build on the Intervention-Context-Mechanism-Outcome configuration of the ART adherence club intervention in Western Cape [25] to develop a programme-specific CMOc and IPT for the KP-CBART in Benue State, Nigeria.

### Step 6—data analysis and synthesis

Thematic content analysis [28] was used. Interviews with the programme managers were recorded, transcribed and uploaded into the Nvivo 12 software R1.6.1 (1137) for coding and analysis. The first author and a research assistant transcribed the audio recordings. The uploaded transcripts were read and coded to identify the likely contextual factors, mechanisms, actors, and outcomes of the programme. We complemented Nvivo data analysis with data abstraction and analysis in an excel-based data template.

To obtain plausible explanations for how KP-CBART is expected to work in Benue State, Nigeria, we triangulated data and synthesised findings using the heuristic tool to configure the CMO for KP-CBART from different sources. First, we identified the different attributes of CMO and then through the process of configuration mapping “– an approach to causality, whereby, outcomes are considered to follow from the alignment of a specific combination of attributes” [14, 25], we aligned a combination of attributes. This process of configuration was achieved using the logic of retroduction and abduction. Retroduction refers to asking why things are being observed as they seem to be. Retroduction is the identification of hidden causal forces that lie behind identified patterns or changes in those patterns [29, 30]**.** Using the CMO heuristic tool [14], we extrapolated the contexts, mechanisms, and outcomes.

Table 5 below provides an overview of the key themes for the data analysis. We distinguish in context between micro, meso and macro, considered here as individual, organizational, and societal factors [31] and in outcome, between early, intermediate, and long term outcomes.

**Table 5:** Definition of terms

	**Definition**
Intervention	HIV service delivery models for key populations in ta specific community setting
Context	The existing setting (environment) into which the KP-CBART programme is introduced and the interaction with existing policies, procedures, attitudes, and beliefs and priorities
Micro (individual-cogntive)	Individual and inter-personal related factors such as sexual orientation, age, socioeconomic status, etc
Meso (relational-organisational)	The service delivery areas (i.e. ART centres, outreach venues, DIC) and the community in which the different service delivery points are situated
Macro (societal)	The overall factors, for example, the state or national level i.e. hostile legal/policy environment for example 14 years imprisonment for same-sex offense, harassment by police, stigma and discrimination
Actors	Institutions or agencies, population groups and individuals who play a role in the KP-CBART such as medication adherence counselling, ART refill
Mechanism	The underlying entities, processes, or structures which operate in particular contexts to generate outcomes of interest [32] The way in which a programme’s resources or opportunities interact with the reasoning of individuals and lead to changes in behaviour (‘response to resources’) [14]
Outcome	A change that is caused or created by the KP-CBART programme
Early outcome	The immediate effect of the KP-CBART
Intermediate outcome	The indirect effect of the KP-CPART programme activities
Long term outcomes	Changes at micro, meso or macro level in the the longer term as a result of the CBART intervention e.g. change in national HIV/AIDS guidelines

The CMOc were extrapolated from each document reviewed and interview, and patterns across data sources (demi-regularities) were identified to inform the IPT. Thereafter, we built on the work of Mukumbang et al. [22, 33] that identified most of the mechanisms and contextual factors influencing outcomes among PLHIV and using the generative causality logic (retroduction), we developed CMOc that explained the relationship between the different types of CBART interventions, contextual factors, actors, mechanisms and outcomes.

### Formulating the empirically testable theory

We identified testable programme theory by translating the CMOc using *the “if, then, because statement”.* Alternate programme theories were formulated from the various identified testable theories.

## Results

The results section is presented in two parts. The first part describes the CMOc that are key to the success of the KP-CBART implementation based on the IPT developmental stages described above in the methods section. The second part describes the IPT of the community-based ART programme for members of KP in Benue State.

### CMO Configurations

**Fig. 4:**
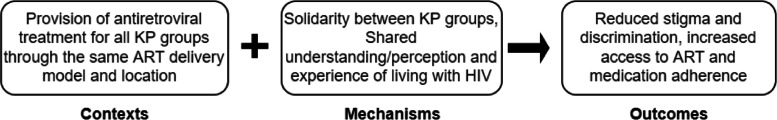
CMOc 1 (KP- key populations, ART-antiretroviral therapy)

**Fig. 5:**
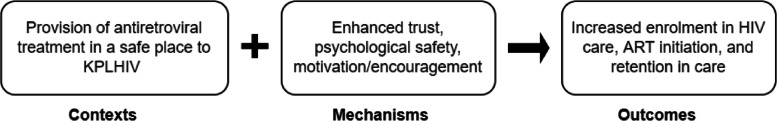
CMOc 2 (*KPLHIV-Key population living with HIV, ART- antiretroviral therapy*)

The various interacting and interdependent factors within the KP-CBART programme were identified during the IPT development stages and categorized into CMOc (supplementary tables 4 , 5, 6). Seven [7] of the CMOc were prioritized and explicated in this section. Figures. 4 and 5  demonstrate the interaction between and within the different contexts influencing the various programme outcomes and causative mechanisms.

Table 6 depicts the patterns of CMOc observed across studies and implementation guides during the desk review and the in-depth interviews with programme managers of the KP-CBART. Thereafter, a narrative on each of the CMOc is provided which include the data sources and illustrations of citations backing up the configurations (thus identified).

**Table 6:** CMO Configurations

CMO configurations	Context	References	Data source
CMOc 1: Solidarity between KP groups (Fig. 6)	*If* community-based ART model is designed to provide ART to all the 4 KP groups (MSM, FSW, PWID, & TG) in the same location (**C**), *then* members of the various KP groups will not stigmatise and discriminate one another (either within or between KP groups) (**O**) and will have unhindered access to ART (**O**) *because of* the shared understanding (**M**) and perception that all KPs are at substantial risk of HIV infection and victims of criminalization laws, stigma, and discrimination in the community regardless of their group (**M**). *This can result in* solidarity (**M**) among KP individuals leading to better engagement with the HIV care and treatment programme (**O**)and cost savings (**O**)	Nil	Interviews: IDI—1 & 5)
CMOc 2: KP friendly environment/Safe space (Fig. 7)	*If* Comprehensive HIV services are provided to members of KP in a conducive environment that is safe, non-stigmatizing, non-discriminatory, and friendly (**C**) t*hen a*ccess to antiretroviral treatment (**O**) and adherence to drug and/or clinic appointments (**O**) will improve *because t*hey (KP) feel safe- psychological safety (**M**) and have trust (**M**) in the programme and healthcare providers. Also, privacy and confidentiality are preserved *As a result, *KP are motivated (**M**) and encouraged (**M**) to remain in care (**O**) and to achieve optimal viral load suppression (**O**)	[34–37] [38–40] [41] [42] [43] [44] [45] [19]	Interviews:IDI-1–9, IDI-11, 12, 13) 4 peer reviewed articles, 4 guidelines, and 2 programme reports
CMOc 3: Communty ART outreaches to address geographic and structural barrier to ART (Fig. 8)	If KP individual could receive ART through community ART team in hotspots or outreach venues in locations where there are no OSS or DIC (**C**) then KP will have early accesss to ART(↓transportation cost, travel & waiting time)- (**O**), clients satisfaction, increased ART uptake and medication adherence (**O**) because of the level of trust in the expertise of cART team (**M**), the quality of care provided (**M**), psychological safety (**M**), and feeling of self-importance (**M**) Resulting in improved retention in care, viral load coverage and suppression	[38–40] [41] [42] [43] [19] [46, 47]	Interviews: IDI 1 – 5, 7- 9, 12, 13 4 peer reviewed articles, 3 guidelines, and 2 programme reports
CMOc 4: Peer support through participation in community support group meetings (Fig. 9)	*If* KP individuals actively participate in support group meetings through peer education and interpersonal communication (also benefits from ART refill and viral load sample collection during meetings) (**C**) *then* awareness and knowledge of HIV/AIDS will increase **(O)** and a change in attitude towards medication adherence (**O**) *Because of* group learning (**M**), Group identity (**M**), -Mutual support/solidarity (**M**) self-efficacy (**M**)*,* *Resulting in* improved medication adherence, retention on ART, and viral suppression	[48][40] [41] [42] [43]	Interviews: 1DI- 1,2, 4, 6, 8, 9, 10, 11, 13 1 peer reviewed article, 3 guidelines, and 1 programme report
CMOc 5: KP community engagement and participation (including peer support) (Fig. 10)	*If* KP individuals (**A**) are actively engaged to participate in the planning and implementation of HIV services (i.e. outreach planning, peer support, HTS, ART refill) **(I)** – in the community-based ART model for KP (**C**) *then* ART uptake and medication adherence will improve **(O)** *because* of meaningful KP community participation **(M)**, privacy and confidentiality**(M)**. KP clients will develop trust in the healthcare providers **(M)**, feel safe **(M)**, and buy into the programme **(M)** *As a result,* KPs will perceive services to be KP friendly **(M)** and they are motivated and encouraged **(M)** to remain in care (retention in care) **(O)**. Thus, achieving optimal viral load suppression **(M)**	[34, 36] [38–40] [41] [42] [43] [44]	Interviews: IDI-1–9, IDI-11 3 peer reviewed articles, 5 guidelines, and 2 programme reports
CMOc 6: Capacity building and technical support/mentoring and supportive supervision (Fig. 11)	If continuous training and mentoring (KP sensitization, HIV case management and comprehensive ART training) in the KP-CBART programme are offered to the heathcare providers (lay health workers and professional medical staff) and law enforcement agents **(C)** then this will increase awareness and knowledge among providers’ knowledge, a change in attitude towards KPLHIV and Healthcare providers will provide culturally sensitive and appropriate HIV services **(M)** to members of KP **(A)** because they are empowered **(M)** to provide quality HIV services to KP individuals (self-efficacy) **(M)** Resulting in improved ART uptake **(O),** medication adherence **(O), and reduction of r**educed stigma and discrimination **(O)**	[35, 38–40, 49] [41] [42] [43]	Interviews: IDI 1 – 10, 13) 3 peer reviewed articles, 4 guidelines, and 2 programme reports
CMOc 7: Programme ownership and sustainability (fig. 12)	*If* the CBART programme (actors) engages with the key stakeholders (such as the policy makers, law enforcement agents (government), HIV Agencies, KP networks, and PLHIV network) through advocacy and sensitization about the KP programme **(C)** *then there will be* increase in awareness and knowledge of HIV **(O),** and *a c*hange in attitude towards of KP **(O)** ***,*** **b** *ecause stakeholders buy into the programme* Resulting in programme ownership and sustainability **(O),** *f*ormulation of KP friendly policy implementation **(O), and reduction in stigma** and discrimination /harassment by police **(O**), and prioritization of HIV programme for KP **(O)**	[38–40] [43, 44] [41] [42]	Interviews: IDI-1, 4, 7, 8, 10, 12 4 guidelines and 2 programme reports

*KI* Key informant interview, *DR* Document review

**Fig. 6:**
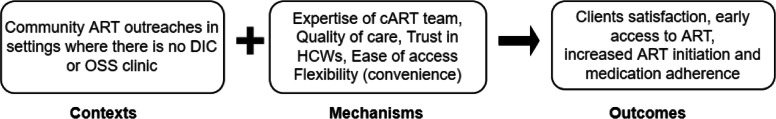
CMOc 3 (*cART-mobile ART team, HCW-healthcare workers, OSS-one stop shop, DIC-drop in centre, ART-antiretroviral therapy*)

**Fig. 7:**
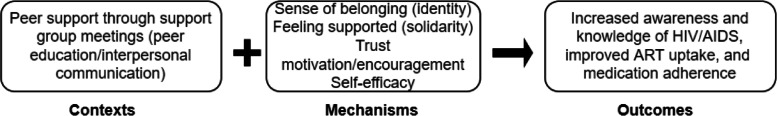
CMOc 4 (*ART-antiretroviral therapy*)

**Fig. 8:**
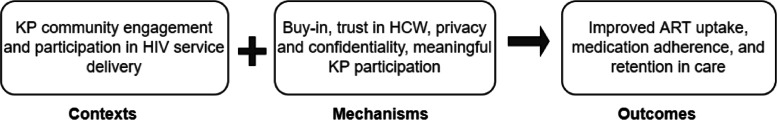
CMOc 5 (*KP-key population, HCW- healthcare workers*)

**Fig. 9:**
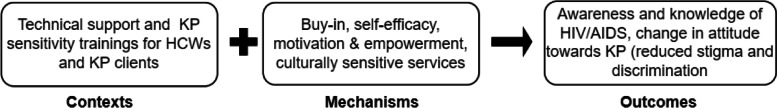
CMOc 6 (*KP-key populations, HCW-healthcare workers*)

**Fig. 10:**
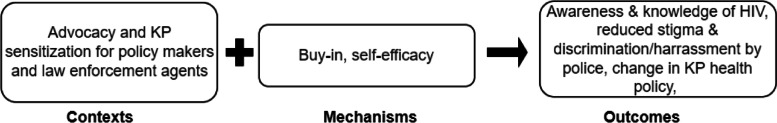
CMOc 7 (*KP-key populations*)

**Fig. 11:**
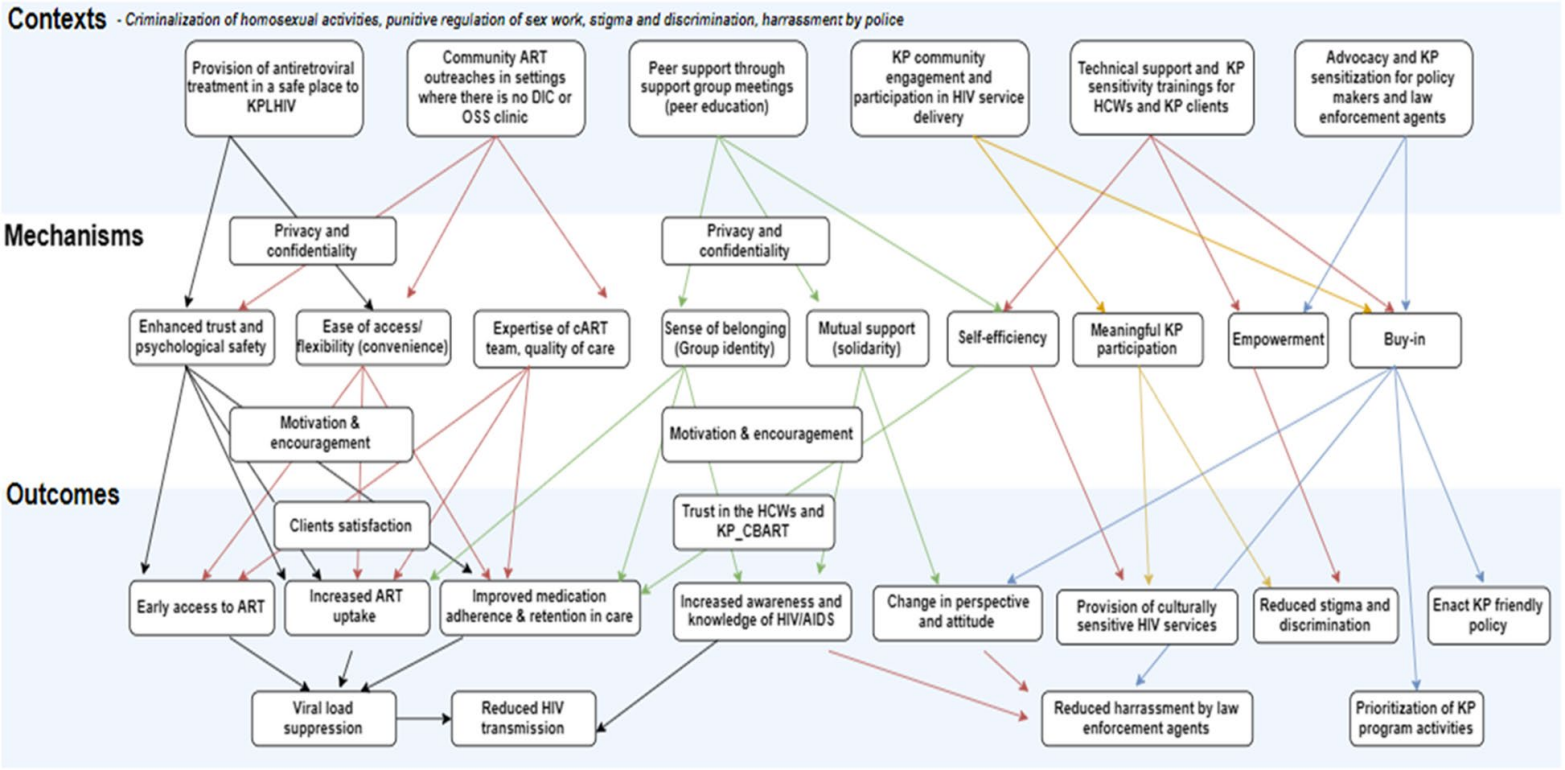
CMO Configurations depicting the Initial Programme Theory of the community-based ART models for KP in Benue State, Nigeria (Priority CMOc based on document review, scoping review, and interviews)

**Fig. 12:**
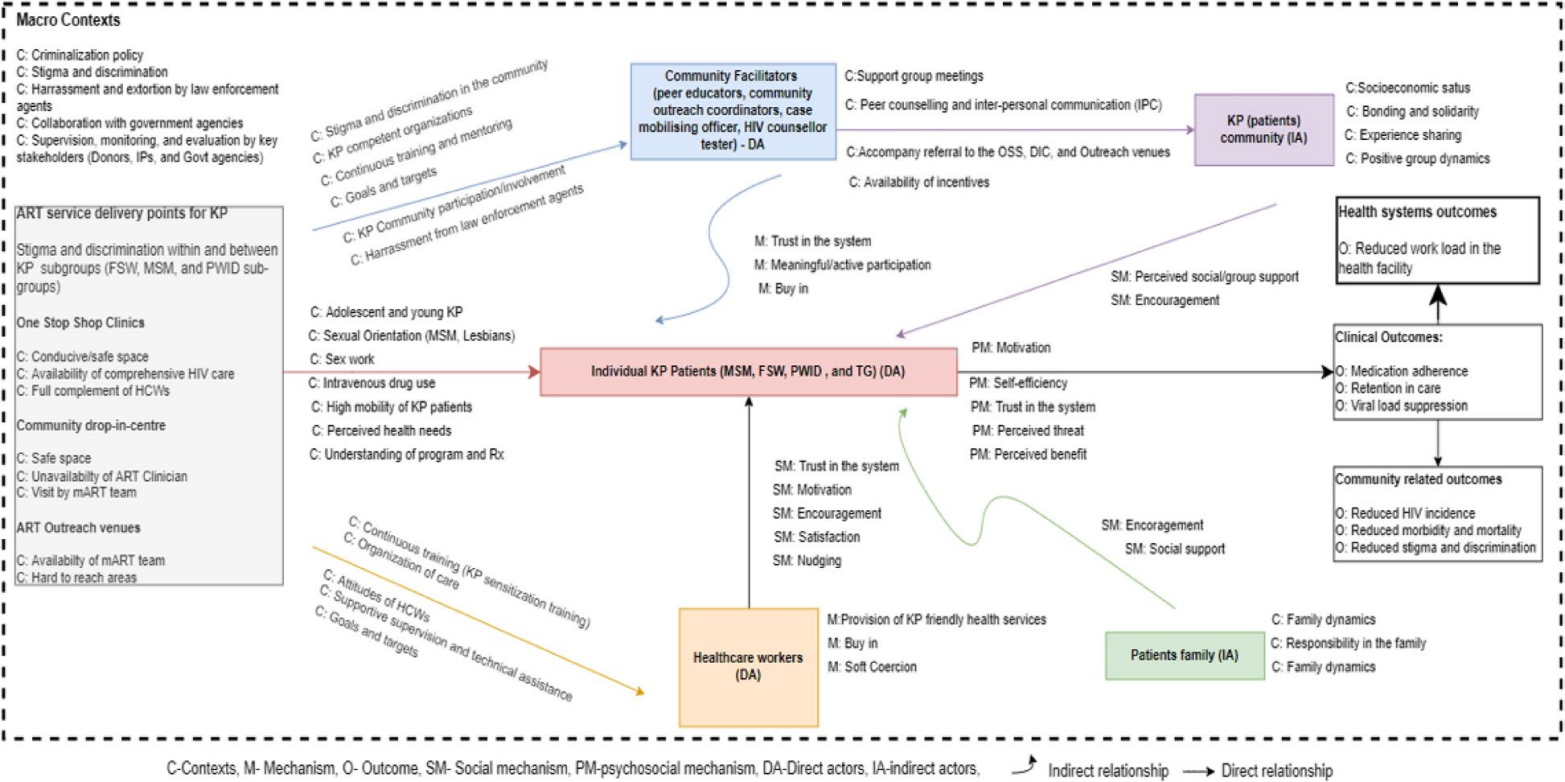
The IPT for the community-based ART models in Benue state, Nigeria (based on the existing theory of adherence club in Western Cape, South Africa)

There was no national health policy or guidance that recommended a preferred model of ART service delivery for any of the KP subgroups in Nigeria or elsewhere. Most KP-CBART model in Sub-Saharan Africa targeted a specific KP group. In Benue state, Nigeria, programme managers reported that the KP-CBART model was structured to provide ART to all the KP sub-groups (FSW, MSM, PWID, and TG) in the same model and location. Funders and partner organizations perceived that all KP individual or groups were at high risk of HIV and were equally stigmatized and discriminated against in the Nigerian society (C). Therefore, they reasoned that if a community-based ART model provided ART to all of the four KP groups (MSM, FSW, PWID, & TG) in the same location (I), then members of KP would not stigmatise and discriminate one another (either within or between KP groups) (O) and their access to ART will not be hindered (O) because of the shared understanding (M) and perception that all KPs are at substantial risk of HIV infection and victim of stigma, and discriminaton in the community regardless of group identity (M). This can result in cooperation and solidarity (M) among KP individuals leading to better engagement with the HIV care and treatment programme (O) and cost savings (O).

*“In order not to waste scarce resources and further stigmatize or divide members of key population, I think that is why ART services are provided through the same model of service delivery and in the same location. This arrangement of HIV care for KP will increase their access to HIV prevention and treatment services and it will encourage tolerance and collaboration among the different KP groups. It provides opportunity for FSW and MSM living with HIV to collectively make demands and support each other treatment’’,* ***Programme Manager***

*“…there is really no positive or negative effect of having members of all the KP groups receive HIV treatment in the same community-model of ART. I have not heard report of violence or any issue arising from stigma or discrimination between members of the different groups’’,* ***Programme Manager***

In a setting with an unfavorable policy against KP activities and stigma (C), all the interview respondents were of the opinion that the most important enabler of the KP-CBART model was the provision of safe space for antiretroviral treatment (I). A safe space is a conducive environment for KP that is non-stigmatizing and non-discriminatory. Examples of safe spaces for KPLHIV in the Benue State CBART programme include OSS clinics, DICs, and OVs.

Many KP individuals tend to be afraid and hide their sexual orientation and HIV status in a context where there are punitive laws, stigma and discrimination. This type of context could serve as a barrier to linkage to ART and retention in care. Evidence from peer reviewed aricles and interviews of KP programme managers showed that a safe place (KP friendly environment) can enhance KP clients’ trust and psychological safety in the programme and healthcare providers. These mechanisms motivated and encouraged KPLHIV to initiate ART and adhere to clinic and drugs refill appointments in KP-CBART.

*“ ……they (KP individuals) respond to services that are being provided because these services are trusted,* ***confidential,*** *and readily available. So that is what drives the response from the KP community. Once they are able to trust the service and they are sure that everything that happens there is confidential and in particular see that it is a safe place…” Programme Manager*

*“….. these clients (KP) don't get to access the regular health facilities where other people go because they are* ***afraid of being stigmatized*** *because of who they are, or what they do. So they do not access services (HIV treatment), thereby creating a gap. So these services need to be taken to where they live, and where services can be provided to them (KP) in a non-judgmental way and where they can easily access it without being stigmatized.’’ Programme Manager*

*‘’..In Nigeria, community-based HIV services for KPs are offered through one-stop shops to meet their comprehensive prevention, treatment, and care needs. One-stop shops are* ***“safe spaces*** *” where KPs receive non-discriminatory services. They require less time and travel expenses than secondary and tertiary health institutions, minimize stigma, and promote quality care in a culturally sensitive manner’’ National HIV guidelines Pg 160,* [19]

The 3 OSS clinics and 2 DICs serving KPLHIV were inadequate to meet the HIV treatment needs of KPLHIV in Benue state Nigeria. KP individuals residing where there was no KP friendly health facilities or in hard to reach areas (due to communal clashes and farmer-herders crisis) experienced difficulty accessing quality HIV care services (C). This setting presented both structural and geographic barriers to ART. Therefore, mobile CBART outreaches for ART initiation and refill (I) was designed by the programme to increase access of KPLHIV to ARVs (O) by removing the barrier of high cost of transportation, long travel and waiting time to the available HIV treatment centres. mobile ART team consisted of ART clinician or nurse, lay adherence counsellor, and pharmacist. Clients’s trust in the KP-CBART (M) and healthcare providers, the ease of access to ART (M), the expertise of the mART team (M), and the quality of HIV care (M) provided during ART outreaches were some of the factors that motivated (M) and encouraged (M) KPLHIV to initiate and remain in care. The quality of care obtained/received during ART outreaches was said to be comparable to that of KP friendly health facilities [13].

*“….. not all the key population patients will be able to come to the facilities to receive care and KPs being highly mobile individuals are not always in a specific locality at every point in time. So the community based ART model is to ensure that you take HIV services to them (KP) and that way you eliminate costs, eliminate drop off in treatment, and ensure quality care. …Also, you know that KPs don't want to go to the health facilities for general population, even if you refer them… why? because of the stigma associated with KPs. So, the community-based ART outreach helps to reach out to them where there is no OSS ..“* ***Programme Designer.***

*‘’…..Okay, the community ART, yes it benefits the individual patient because we're bringing the services to you at the same quality that you would get if you are coming to the OSS clinic, so we cut off transportation risks, transportation time for the client, okay, ..so the client has more time to do other personal things which they want to do, make other time investments* *,* *and then just come out at a specific time four to five hours when the team is available, and receive their medications, get tested for viral load, and leave so it's quite impactful so that way they can easily maintain their businesses run their businesses and still get their treatment without compromising’’.* ***Programme Manager***

In a setting with an unfavorable environment (in terms of punitive laws, criminalization policy and arrest by police) against KP activities (C), Support group meetings (SGM) (I) was one of the key implementation strategies for stigma reduction (O) and clients’ medication adherence (O). Community-based SGM were organized monthly or quarterly depending on the resources available in the programme. During the SGM, KPLHIV were mentored and coached by peers through peer education and interpersonal communication sessions. Participation in SGM increased the level of awareness and knowledge of HIV/AIDS among KPs (O) and triggered a change in attitude—medication adherence and retention in care (O). Through experience sharing and (problem sharing/solving), mutual support/solidarity and group identity (a sense of belonging (solidarity), clients would develop confidence and trust in themselves (self-efficacy) and were empowered to care for self (M) – to initiate and adhere to ART (O).

*‘’….we also hold support group meetings, that also help other persons who are experiencing/ passing through issues. People are wired differently, some persons may be getting their ART treatment and begin to find some issues with taking the drugs like drug reactions and all of that. And so when things like this happen, they are best resolved in support group programme, where people share their experiences, and other people to encourage others on how to remain positive. And all of that we do all of that….’’,* ***Programme Manager.***

*‘’…. the support group meetings, it's one of the greatest strategy because it makes them (KP) to interact among themselves, get to talk to other persons that are also infected with HIV and AIDS, where everybody's talk about their experience, their challenges, and then how best they could handle that. Most of the time they see that it is not just me, they see that other persons are also involved in this. So it gives them that confidence to be able to talk and interact with them and they are open to discuss’’,* ***Programme Manager.***

*“ Yes, it (support group) has worked because usually during providing orientations for them or providing these services.. they are brought together, and it makes you ooh I'm not alone. This person is also affected…this is because they all come to the same place. And it also helps to also provide them a safe house for them because you have somebody you can talk to so that trust and over time the key population has realized they've been able to trust the providers of these KP services. And there's this positive collaboration between the service providers and the clients.’’,* ***Programme Manager***

In a setting of punitive laws against KP and discrimination (C), the engagement of KP individuals to actively participate in programme planning, implementation, and evaluation (I) was a key intervention that influenced outcomes in the KP-CBART. In the KP-CBART programme, KP individuals were engaged and trained by KP competent CBOs as lay HCWs (as peer educators, case managers, outreach coordinators). These lay HCWs (KPs) were empowered through training, and other capacity building interventions such as mentoring and technical assistance.

*‘’…One of the most important and single achievement of the KP-CBART* ***is empowering the community*** *through their meaningful involvement in HIV programme implementation. …..having to empower the community with skills set for implementation, monitoring and evaluation, ……and all of that. ……should I say, it (community participation) would make our programme to outlive our strategies….’’,* ***Programme Manager.***

*‘’For me, we have given them (KP) an opportunity to express themselves by involving them as either* ***peer educators,*** *community facilitators, community testers, and all of that, when you involve them, you notice that it improves their self-esteem. ……..And then from from interacting with you (programme officers/maangers), they also begin to learn values from implementing…. ..in our state practically I've seen girls that we pulled out from brothels go back to school without you telling them but from their roles as community facilitators, they will see that aah they will need to build their skills for documentation and all that’’,* ***Programme Manager.***

KP community participation in HIV service planning and delivery (as lay health service providers) (I) could trigger a sense of meaningful participation and self-importance in the programme. This would enhance KP’s trust in the KP-CBART model because members of the KP community connected and provided services that were tailored to individual KP health needs. Members of KP were better positioned to provide private and confidential HIV services to peers compared to non-KP. KP bought into and took ownership of the KP-CBART programme, resulting in increased ART uptake, medication adherence, and retention in care.

*“participation is the active involvement of key population members in the planning, design, and implementation of programs. Meaningful participation of key populations is essential to building trust and establishing relationships that will make programs effective in the long term. Participation is meaningful when key populations choose how they are represented in the process of planning and designing programs, and who will represent them. It also means that their opinions, ideas, and contributions are given equal weight alongside those of people who are not key population members.”,* ***Linkages- KP Implementation Guide for HIV care, prevention and treatment***

*‘’Haa wow, it was like aah you people want to come and carry us and go and report us to the police that this is where they are staying now? We say no* ***, we are FSW like you but*** *we've come out now so that we can help each other. So, when they saw our consistency, they saw how we go to them and we let me use this word we ‘roll together’, we go to night clubs together, we do business together they now said ooh these people are same people with us so we shouldn't be scared of them and they became open to us’’,* ***Programme Manager.***

*‘’So from interaction with them they are happy, one because they can walk into the center and be sure that whoever is going to provide service to them is going to be a non-judgmental thing, so they are comfortable with that setting and they know that whether I have an appointment, whether I don’t have an appointment I can decide to walk in there and want to see somebody and other services could be offered to me. So the comfort they have when they stay there is something that has really boosted the program, they also see it as their own because they associate with it because some key population members work within those facilities and so they see it as their own kind of place’’* ***Programme Designer.***

In a setting with an unfavorable policy environment (in terms of punitive laws, criminalization policy and arrest by police) against KP activities (C), health care providers in the KP-CBART received training on various aspects of the programme to build their capacity to deliver high quality HIV services (I). To ensure that healthcare providers provided KP friendly health services (O), they underwent KP sensitization training. The programme partnered with law enforcement agents (i.e. Nigeria Police Force) and provided KP sensitization training to them. Other trainings for heathcare providers include comprehensive HIV care and treatment, syndromic management of STI, and responding to gender based violence. Evidence suggested that capacity building (including KP sensitization trainings, mentoring and supervisory activities) is associated with increase in awareness/knowledge of HIV/AIDS (O) and can stimulate a change in attitude. These encouraged stakeholders to buy into the programme and they provided culturally sensitive and quality HIV serives to members of KP.

*“ ….One of the successful strategies is that the program has been* ***able to build capacity of health care*** *workers in terms of providing services to key population in a non-judgmental way and that they (KP) also have rights to assess health services… you know, we know that the law.. it's a bit not to their favour but they have their human rights, they also have to access care and ….. the program has helped to bring ART services closer to them and a lot of them have been identified and have been placed on treatment and they are stable, they are virally suppressed, and are also reducing the rate of new transmission among them (KP) and within the community.’,* ***Programme Designer.***

*’’We(HIV/AIDS Agency)) are involved in training for healthcare workers, in also providing supportive supervision to these facilities, and then also to engage with other relevant stakeholders to bridge the gap between the partners that are providing these services and other government agencies to be able to provide enabling environment for service provision within these communities, so we've been involved in trainings, in stakeholder engagement and also assessing the positive impact through supportive supervision to see to also have a feedback from the people that are also assessing these services’’,* ***Programme Manager.***

In contexts with a policy that criminalizes KP and in an environment with prevailing harassment from law enforcement agents (i.e. Police), members of KPs hid themselves and were hard to reach with health intervention due to fear of stigma, discrimination and arrest. Many KP felt the KP-CBART programme was a bait to lure them to the open and to prosecute them. To address this issue, advocacy and KP sensitization trainings were designed and planned for policy makers, HCWs and law enforcement agents to increase their level of awareness and knowledge of HIV/AIDS and to stimulate attitudinal change towards KP. Helping the policy makers, local authorities, other stakeholders such as law enforcement agents, police officers (and health programme managers) to understand the goals and objectives of the KP-CBART could encourage the policy makers to buy into the programme and to prioritize health interventions for KP. The programme partnered with law enforcement agents (i.e. Nigeria Police Force) and provided KP sensitization training to them. Policymakers and law enforcement agents (i.e. policemen, civil defense) will be motivated to enact KP-friendly health policies towards increasing access to ART and to reduce community stigma and discrimination.

*“We did an advocacy to the police and as a matter of fact the police are standing with us, we have a good relationship with the gender focal person in the police department and we have a good relationship with the lawyer. We have a lawyer that is attached to us that carry out this activity that goes out standing for our peer group, our community members.”,* ***Programme Manager.***

*‘’ We did an advocacy to* ***the local authorities to avoid*** *stigmatization because that is one thing we as community members we are fighting against discrimination and stigmatization, so we did an advocacy to them and then we tell them that this is what we are coming out for. And we were welcome because you can't just leave your place and go to a place and then you'll be carrying out activities there you need to carry out an advocacy to the various stakeholders in that community’’* ***, Programme Manager.***

*“Due to the hostile and nonpermissive environment as evidenced by the Same Sex Marriage Prohibition Act and other constraints, the continuous and multi-level advocacy with* ***government and non-government stakeholders will be prioritize*** *d.”* ***PEPFAR Country Operational Plan (COP) 20.***

*‘’But of course, having to meaningfully engage with… different stakeholders , we identified stakeholders and we sensitized them. Stakeholders like the police force, the legal authorities, the legal entities, the duty bearers… basically, the custodians of those laws that have been set. We engaged them and we sensitized them of the public health benefits of having to intervene with this group. So initially, it wasn't but as we sensitized them and as we continued to engage them and carry them along in our programme implementation, we gave them information and provided feedback on how we're faring on the project. It became easier for us to implement within the state’’* ***Programme Manager.***

### The Initial Programme Theory of the KP-CBART

In a setting with an unfavorable policy environment (i.e. punitive laws, criminalization policy and arrest by police) against KP activities and low levels of trust between healthcare workers and members of KP (C):

1. Decentralisation of ART service delivery to KP communities together with training of HCW and law enforcement agents on KP sensitization and comprehensive ART, and advocacy to policy makers will enhance trust (M) and psychological safety (M) in the programme and encourage (M) KP to access HIV care and treatment services and this will improve uptake and utilisation of these services and retention-in-care (intermediary outcome). Optimal HIV prevention and treatment for KP will translate to better health outcomes and well-being for KP (final outcome).
2. Where the KP community and lay health workers are involved in the design, planning, and implementation of HIV services (e.g. accompany referral for ART, medication adherence, ART refill, clients tracing and etc.) (I), KP clients will be encouraged to buy into the KP-CBART because they trust the healthcare providers and perceive HIV services to be KP-friendly (M) and thus, resulting in sustained engagement of KPLHIV in HIV care and clinical outcomes (i.e. medication adherence and retention in care).

## Discussion

This paper expanded on our understanding of CBART for KP, a type of differentiated care, by explaining how and why the different types of KP-CBART models in Benue State will produce outcomes along the cascade of HIV treatment. The aim of the study is not to determine the effect of the KP-CBART on patient outcomes but rather, to explain how the KP-CBART will achieve the intended (or not intended) outcomes, for whom the KP-CBART works and why it works. The IPT was developed relying on various sources of data to describe the contextual factors influencing the KP-CBART, the causal mechanisms, and the programme outcomes. We described the outcomes of various processes that culminated in the IPT of the KP-CBART programme as it is being implemented in Benue State, Nigeria. Also, we identify and prioritize seven CMOc that depict the IPT of how KP-CBART will achieve its objectives of improving ART uptake, medication adherence, retention in care, and viral load suppression.

The IPT indicated that the KP-CBART was designed to provide a safe space for ART delivery to KPLHIV in a setting with punitive laws, stigma and discrimination against members of KP. The KP-CBART model will encourage medication adherence and retention in care because KP will feel safe and trust the healthcare providers. And that the involvement or participation of KPLHIV in the design, planning and implementation of HIV services will improve medication adherence and retention in care because they perceive HIV services to be KP-friendly and participate in KP-CBART. This theory aligns with the World Health Organization and national HIV programmes recommendation for community-based ART delivery for KP and KP community participation in HIV service delivery in SSA [4, 19]. Findings from various studies in sub-Saharan Africa have shown that KP community involvement in HIV prevention and care services has the potential to improve treatment ouctomes such as medication adherence and retention in care among KP [12, 13].

This research demonstrates the significance of a safe and conducive place for KP to initiate and refill their drugs in the KP-CBART programme. All the KP programme managers (interviewees) attributed the success of the programme to the provision of safe space, such as DIC, OSS, and OV, where KP feels safe from the outside world. This safe place offers privacy and confidentiality, and it triggers trust in the programme and the the HCWs. Studies have demonstrated the effect of KP-CBART and safe space on access to ART and clinical outcomes in a context of punitive/criminazing laws against homosexuals and sex workers [13, 47, 50]. These studies showed improved treatment outcomes in different settings in SSA countries such as Tanzania, Zimbabwe, Congo, Nigeria, and Benin. In these settings, ART services were provided in a safe place, such as FSW or MSM specific-research clinic, community and home-based ART delivery platforms [13, 46, 47, 50, 51]. Safe space will trigger the mechanisms of trust and psychological safety (feeling of safety and comfort) in KPLHIV. Also, the level of quality of care and technical competence will motivate and encourage KP individuals to access and remain in care.

The activities of the mobile community ART team in settings where there are no KP friendly health centres have the potential to increase engagement of KP in the programme and to improve medication adherence and retention in care [46, 47]. Mobile ART team brings treatment to where clients live and provide ART in a safe space (outreach venue of hotspots), thereby increasing access to ART. A RCT study in Tanzania evaluated the effect of CBART on treatment outcomes among FSW and findings showed an improvement in retention in care and viral load suppression. Also, studies that summarised the performance of CBART programme among the general population in SSA showed that community based ART including mobile ART outreaches have the potential to improve treatment outcomes among PLHIV in low resource settings [52, 53]. The perspective of the programme managers of the KP-CBART in Benue is that the quality of care and competence of the mART team can motivate and encourage KP clients to access ART and remain in HIV care.

The level of trust in the health care providers (and in the programme) and psychological safety are the key mechanisms that will trigger a change in attitude and high uptake of ART and improved medication adherence. There tends to be an emphasis on safe space and KP community involvement in HIV services delivery (the design, implementation and evaluation of CBART) in the development of trust and buy-in of the KP-CBART by KP clients by the programme managers. When KP peers are engaged to work as lay HCWs, they are able to provide services (such as HIV counselling testing, ART referral and refill in the community ART centres and during outrearches and support group meetings) that are tailored to the needs of KP individuals. These contexts of KP community mobilisation, engagement, and participation will trigger trust and psychological safety, foster mutual learning and solidarity that may lead to change in attitude towards ART uptake and medication adherence. Many studies have examined health outcomes associated with patients’ trust in physicians [54, 55]. A study by Pearson et al. (2000) argued that trust is one of the central features of patient-physician relationships [56]. This study/paper discussed the current theories about trust and empirical data on patient-physician trust. Findings from the study, revealed that patient trust reinforced the functioning of the clinical relationship between healthcare providers and patients, thereby increasing the probability of patient satisfaction, treatment adherence, and improved health status [56].

We could not identify sufficient evidence to explain why the KP-CBART is organized to provide ART to at least 3 different KP sub-groups in the same service delivery point in the community. The rationale for organizing ART care in the same service delivey point through the KP-CBART were not discussed in the guidelines and policy documents for the implementation of HIV care and prevention interventions for KP [41, 43]. However, programme managers believed that the different KP groups do not discriminate among themselves because they all face the same issues in the society. The programme provides an opportunity for the different KPs to meet and to foster unity, solidarity, and collectively pursue changes that can influence their treatment outcomes and well-being. Although KP sub-groups share similarities in terms of exposure to HIV risk and stigma, they are different in terms of sexual orientation, and health needs [57]. We will explore the interaction between and within each of the KP-sub-groups in the KP-CBART to refine this CMOc in the second phase of this realist evaluation.

Several studies have demonstrated the negative effects of punitive laws and highly stigmatized environment and access to HIV treatment and care for KP [58, 59]. A study conducted in Indonesia revealed that PLHIV were reluctant to access healthcare services at the same healthcare facilities and communities where they experience stigma and discrimination [9]. Therefore, there is need for sensitization and training on KP-related topics, including their health needs, and advocacy to policymakers, law enforcement agents, and other stakeholders to decriminalise or reverse the punitive laws against members of KP [60]. When capacity of HCWs, law enforcement agents, and law enforcement agents is developed through sensitization and advocacy, and trainings on health needs of KP in the KP-CBART programmes, then stigma and discrimination may reduce in the community because of the change in attitude towards KP. Where laws penalising the acts of KP are decriminalized or never existed HIV programme managers and HCWs will be encouraged to provide services that are KP friendly and the KP-CBART is implemented with fidelity to the original design and intent of the programme designers. Police and other law enforcement agents will reduce harrasmment and arrest of KPs. Policymakers will most likely enact KP friendly health policies.

### Strengths and limitations

This is the first study in Sub-Saharan Africa that attempted to evaluate the KP-CBART using the realist research method and to elicit IPTs. Several sources of data and views of stakeholders were considered in the analysis. We systematically reviewed grey and published documents, interviewed experienced programme managers and an existing theory of a similar intervention to develop the CMOc and IPTs.. One of the researchers is an insider whose professional experience of the KP-CBART provided insight and in-depth understanding of the programme and stakeholders. CMOc were discussed and debated among the researchers and in a realist group meeting. Selected programme managers were re-interviewed to narrow the scope of the analysis and to confirm the final CMOc. Triangulation of data from various sources, including the realist group meetining, allowed us to validate findings and provide more robust conclusions.

Another strength of the study is the iterative data collection and data analysis. The IDI guide was piloted with programme managers to adapt it to the input of stakeholders and to ensure the right questions were asked. Furthermore, building on an existing theory provided opportunity for cumulation of knowledge, adjudication of theories, and a better understanding of how to adapt HIV treatment programme and differentiated ART delivery to PLHIV to the specific Nigerian context.

While we aimed for thematic saturation during this study, we were limited by the number of programme managers and designers that were available for interview. However, we ensured that study respondents were individuals who were deeply involved in the programme in terms of design, planning, management, and implementation of the KP-CBART programme. Some of the programme managers and designers were re-interviewed for their opinion on the CMOc and the elicited programme theories.

### Implications of findings

The elicited IPT could be the starting point for realist researchers who are interested in CBART for KP and similar models in SSA. This IPT will be refined in the second phase of the realist evaluation cycle through case studies and mixed research methods. The effect of KP-CBART on treatment outcomes such as medication adherence, retention in care and viral suppression will be explored. Furthermore, the perception and experiences of KP clients and HCWs of the KP-CBART will be assessed.

## Conclusion

In this article, we described the KP-CBART model in Benue State in Nigeria, and explained how it works, why, and what circumstances are expected to generate outcomes. Based on our analysis and findings, we identified and prioritized 7 CMOc for explanation and summarised them into an IPT. Here we have provided theory that where KP living with HIV (KPLHIV) receive ART in a safe place while living in a setting of punitive laws, harrassment, stigma and discrimination, KP will adhere to treatment and be retained in care because they feel safe and trust the healthcare providers. We indicated that the involvement of KPLHIV in the design, planning and implementation of HIV services will improve medication adherence and retention in care because they perceive HIV services to be KP-friendly and participate in KP-CBART.

The IPT represents the first phase of the realist evaluation of the KP-CBART in Benue State. The elicited programme theories will be modified (refuted or confirmed) in the second phase of the realist evaluation to elicit a refined programme theory of the KP-CBART programme. Findings from the next realist evaluation studies will inform the adaptation and scale-up of the KP-CBART programme to better cover the health needs of KPLHIV in Benue State, and may inform CBART programmes in similar settings in Nigeria and SSA.

## Supplementary Information

**Additional file 1: Figure 1.** Prisma diagram of literature search for community-based ART model for keypopulations’ study and selection process.

**Additional file 2: Table 1.** Causal model – Assumptionsof the programme managers and designers - the community-based ART Programme inBenue State, Nigeria (informed by the intervention logic model and professionalexperience of the researcher).

**Additional file 3: Table 2.** Characteristics of papers included in thedesk-review of literature on the implementation of  community-based HIV prevention, care, andtreatment for key Populations in sub-Saharan Africa.

**Additional file 4: Table 3.** Topic guide for in-depth interview with programme designers/managersin the community-based HIV programme for key populations in Benue State,Nigeria.

**Additional file 5: Table 4a.** Intervention and Actors. **Table 4b. **Outcomes.

**Additional file 6: Table 5.** Classification of Mechanisms.

**Additional file 7: Table 6.**Contexts.

## Data Availability

The dataset underlying the results and conclusions of this article are available as part of the article and supplementary tables. If additional information is required, the raw data supporting the results of this study are available from the corresponding author on reasonable request.

## Abbreviations

ART: Antiretroviral treatment or antiretroviral therapy
CBART model: Community-based antiretroviral therapy delivery model
CMOc: Context-mechanism-outcome configuration
DIC: Community drop in centre
FSW: Female sex workers
IBBSS: Integrated Behavioural and Biological Surveillance Surveys
IPT: Initial programme theory
KP: Key populations
KP-CBART: Community-based ART model of service delivery for key populations or CBART for key populations
KPLHIV: Key populations living with HIV
MSM: Men who have sex with men
NAIIS: National HIV/AIDS Indicator Survey
OSS: One Stop Shop
OV: ART outreach venue
PLHIV: People living with HIV
PWID: Persons who inject drugs
TG: Transgender people
