# Supplementary material for: Initial programme theory for community-based ART delivery for key populations in Benue State, Nigeria: a realist evaluation study
Source: BMC Public Health. 2023 May 12;23:870. doi: 10.1186/s12889-023-15774-w (PMC10176666; doi:10.1186/s12889-023-15774-w)
Supplement: Supplementary file 1 — Additional file 1: Figure 1. Prisma diagram of literature search for community-based ART model for keypopulations’ study and selection process. [file 12889_2023_15774_MOESM1_ESM.docx]

| Supplementary Table 1. Causal model – Assumptions of the programme managers and designers - the community-based ART Programme in Benue State, Nigeria (informed by the intervention logic model and professional experience of the researcher) | | | |
| --- | --- | --- | --- |
| SN | | **Focus Questions** | **Responses (build up to ICAMO)** |
| 1 | What change (outcome) does it intend to create?  *(aim to identify intended outcomes)* | | - Improved linkage to HIV care and ART - Improved retention in care - Improved viral load coverage and suppression rate - Improved access and utilization of HIV prevention and treatment services - Reduced HIV incidence and prevalence among KP (maybe difficult to assess) - Improved health and well-being |
| 2 | Who does it intend to do what differently? (May be several groups).  *(Aim to identify whose decision-making should be examined)* | | **Members of key populations**   - To participate in the provision of HIV service delivery (HIV care and treatment) to peers: - KP opinion leaders, KP networks, PLHIV association/network (NEPWHAN) to participate in programme planning, implementation and evaluation - Mobilizing and educating peers on HIV services (peer educators)- escort services, ART referral, drug refill by proxy, tracking - To engage more with HIV care and treatment (retention on ART)   **Health care workers**   - To change the attitude of HCWs to members of KP: provide KP friendly health services (free of stigma and discrimination) - Stimulate a shift from facility-based care to community-based HIV care: provision of HIV services within the community- OSS, DIC, community outreach   **Other stakeholders**   - Donor agencies, implementing partners, KP-led/friendly CBOs are more responsive to health needs of KP (instead of reactive) - Reduced harassment from the law enforcement agencies - It will ensure govt agencies (MOH, NASCP, NACA) participation for ownership and sustainability |
| 3 | What does it provide to enable that choice or behaviour?  *to identify the intermediary outcomes* | | - Dedicated ART centre for KP, community drop-in centre, outreach venues 🡺 offers privacy and confidentiality🡺 feeling safe & secured, sense of community/ belonging🡺trust in the system🡺 increased service uptake and utilization - KP sensitization training 🡺 buy-in 🡺 change in behaviour and attitude 🡺 reduced stigma and discrimination from service providers 🡺increased engagement in HIV care - Peer-led HIV counselling and testing 🡺 trusting relationship 🡺 buy-in🡺increased service uptake - Drug refill by proxy 🡺 offers flexibility 🡺 triggers feeling of importance/relevance🡺 medication adherence & retention - Provision of KP friendly HIV treatment and wrap around services (STI mgt, condom distribution🡺 additional benefit🡺 feeling important/relevant 🡺 increased uptake and utilization of services - Escort services and tracking of defaulters (by peers & lay HCW) = gently push/encourage patients 🡺 increased retention |
| 4 | How might different sub-groups of staff and participants respond to the resource?  *(to identify the sets of reasoning & resources (mechanisms)* | | **Key populations**   - Community-based HIV care provides opportunity to interact with other members of KP and make friends **(social interaction, social inclusion-cohesiveness, solidarity)** - KPs are motivated to receive ART through the model because they feel safe and trust the system to protect their privacy and keep confidentiality **(Trust and feeling comfortable)** - **KPs helping each other (solidarity, reciprocity)** - **Feeling of distrust** for care providers who are not KP members - **Feeling of discomfort** receiving care in the same model with other member of KP sub-groups (i.e. FSW vs MSM) - **In service delivery points:** - Fear of accidental HIV Status Disclosure and KP status to other people during visit to OSS or DIC (FSW vs MSM)   **HCWS**   - Trained HCWs are more understanding and adapted to specific needs KP and provide KP friendly HIV services - Trained HCW might feel their role is under threat with task shifting to KP (why?) and thus, be hostile to KP lay workers or peer counselors - discriminate and stigmatize? |
| 5 | What features of context affect how people respond to the resources? In what ways do those features affect responses?  *(To identify the contexts- contributes to identifying for whom may/may not work)* | | **Overall political and legal environment**   - Societal stigma and discrimination   **Local context of each SDPs**   - Safety and security - Religious affilitations and posturing   **KP friendly health environment**   - **At service delivery level:** *(nil stigma & discrimination, participation of KP community in HIV service delivery, social networks)* **:** it builds trust in the system and encourages medication adherence leading to good treatment outcomes (viral suppression and retention in care)   - Per service delivery point -specific to drop in centre:   - OSS:   - … - ***Criminalisation of homosexuality and sex work:*** *KP may feel the KP programme is a trap for arrest🡺 distrust and fear of arrest through the programme🡺 poor engagement in care* - **Poverty:** high cost of transportation **🡺 poor adherence** - **Short waiting time:** facilitate ART adherence appointment |
| 6 | Which other features of context will affect whether & how the programme ‘works’?  *(To identify the contexts- contributes to identifying for whom may/may not work)* | | **Location of the community-based ART approach/model:** urban, rural, semi-urban (one model per location)  **Type of community-based organisation:** KP-led or KP-friendly CBOs  **Financing: program ownership, free HIV services**  **Technical assistance from stakeholders**  **Leadership and governance:** management structure of the OSS and DIC, collaboration with KP led and community based organizations  **Information management:** EMR, social media platform, patient biometric system |
| 7 | What outcomes would be generated by different decisions?  *(To identify wider range of potential outcomes)* | | **Decision to enrol in the programme:** increased linkage to HIV treatment, medication adherence, retention in care, virological coverage, and virological suppression.  **Disengagement from the programme:** high attrition rate, poor medication adherence, and treatment failure (non-virological suppression)  Resistance of health workers, both trained and non-trained  Resistance from communities living in the vicinity of the SD point  More harassment from police,  State, local government officials disengage from the programme  Funders no longer support due to difficult environment |
| 8 | What information will be needed and could be collected about contexts? Mechanisms? Outcomes?  *(Required data for analysis/evaluation)* | | Refer to table 2 below:  Contexts: Qualitative data (sources:…) Mechanisms: Qualitative data (sources:…) Outcomes: Tested for HIV, linked to ART, Retention, viral suppression (Quantitative data) |
