# Supplementary material for: Initial programme theory for community-based ART delivery for key populations in Benue State, Nigeria: a realist evaluation study
Source: BMC Public Health. 2023 May 12;23:870. doi: 10.1186/s12889-023-15774-w (PMC10176666; doi:10.1186/s12889-023-15774-w)
Supplement: Supplementary file 2 — Additional file 2: Table 1. Causal model – Assumptionsof the programme managers and designers - the community-based ART Programme inBenue State, Nigeria (informed by the intervention logic model and professionalexperience of the researcher). [file 12889_2023_15774_MOESM2_ESM.docx]

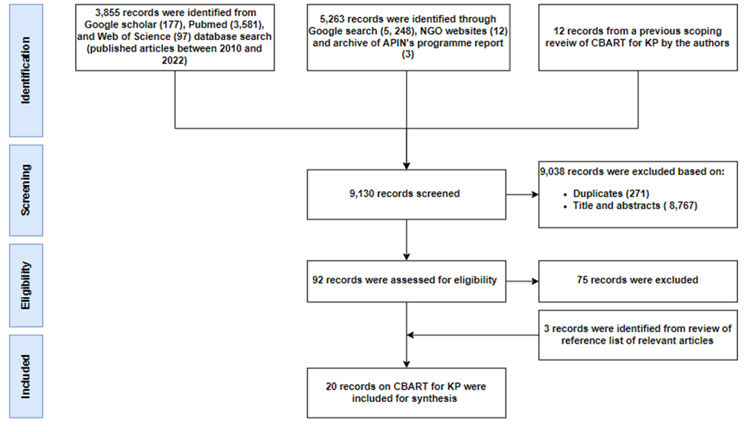


**Supplementary figure 1. Prisma diagram of literature search for community-based ART model for key populations’ study and selection process**
