# Supplementary material for: Initial programme theory for community-based ART delivery for key populations in Benue State, Nigeria: a realist evaluation study
Source: BMC Public Health. 2023 May 12;23:870. doi: 10.1186/s12889-023-15774-w (PMC10176666; doi:10.1186/s12889-023-15774-w)
Supplement: Supplementary file 3 — Additional file 3: Table 2. Characteristics of papers included in thedesk-review of literature on the implementation of community-based HIV prevention, care, andtreatment for key Populations in sub-Saharan Africa. [file 12889_2023_15774_MOESM3_ESM.docx]

**Supplementary table 2. Characteristics of papers included in the desk-review of literature on the implementation of community-based HIV prevention, care, and treatment for key Populations in sub-Saharan Africa**

| **Author name, date** | **Study design** | **Model type** | **Intervention components** | **Reported context conditions** |
| --- | --- | --- | --- | --- |
| Jean Njab et al, 2018 (21) | Observational, descriptive, cross-sectional study | Community-based Test and Start Model for Key Populations through OSS and DIC | HIV Testing Services Same-day ART initiation  STI Management Peer referral for HTS and ART initiation | - Patient sociodemographic status (age, marital status, education) - ART service provision in a safe space  - Distance to the OSS and DIC - Quality of health services - High cost of transportation - Stigma and discrimination - Criminalization and homophobic environment, - Unrestricted access to competent health professionals - Provision of culturally sensitive HIV services - Use of community outreach workers |
| Population Council, 2019 (22) | Prospective cohort study (baseline results) | Community-based Test and Start Model through OSS and DIC | -Conduct community-based HIV testing outreaches  -One Stop Shop Clinic (community-based health centre  -Community drop-in-centre | - High risk sexual behaviour among KP - ART service provision in a safe space that offers privacy and confidentiality to clients - Provision of non-discriminatory and culturally acceptable services to KP - KP commmunity participation in HIV service delivery (recruitment by peers) - Walk-in clients (self-referral to the OSS or DIC) - Stakeholders engagement i.e. quarterly meetings of the Community advisory committees (PACA, CBOs, KP community, HCWs) to address stuctural barriers |
| Afolaranmi et al, 2021(31) | Cross-sectional study | Health-facility based care and community HIV support group | This model targets all HIV‑positive MSM linked to HIV care in any health facility and affiliated to the existing non‑health facility HIV support group | - Having a well established MSM network in the state - Functional KP support groups - Availability of KP friendly healthcare facilities - Stigma, discrimination, violence and criminalization of KP - non-Disclosure of HIV status - Low socioeconomic status |
| Man Charurat et al (2015) (41) | Prospective cohort study | Community-based Test and Start Model | Provision of HIV standard of care through a trusted community centre for MSM  Clinical assessment | - Abscence of protective legislation  - Individual level stigma and discrimination - HCW attitude - Safe space (friendly and trusted atmosphere) - Same Sex Marriage Prohibition Act in January 2014 - Non-disclosure of sexual orientation to both family members and HCWs  - Treatment as prevention  -MSM sensitivity training (culturally sensitive service delivery)  - Standard training in HIV/STI management - Client recruitment through respondent driven sampling |
| Ochonye et al, 2019 (42) | Mixed methods study | Public and peer-led facilities | Provision of peer-led HIV prevention service and treatment. | -Stigma and discrimination -Same-sex prohibition act -Quality of service -Waiting time -Maintaining Confidentiality -Distance to services - Unfriendly appointment hours - Cost of services - Unfriendly and stigmatising health care providers - Staff attitude (stigmatization by the health care workers) - Safe space (privacy and confidentiality) - Fear of accidental HIV status disclosure in the public health facilities |
| World Health Organization, 2016 (3,7) | Guidelines | Community-based approaches to HIV service delivery | -Outreach and mobile HIV services - Drop-in centres and venue-based approaches  - Mobile and/or drop-in services and weekend and/ or night service times that facilitate access.  - Outreach, including venue-based and home-visiting services | - Service delivery are led and delivered by members of the key population community.  - Provision of legal services to reduce stigma and discrimination - Training for health care workers and law enforcement personnel  - Integrated treatment and rights literacy programmes  -Peer intervention /peer-based/peer-driven interventions |
| United States President's Emergency Plan AIDS Relief (PEPFAR), 2020 (29) | Strategic operational plan | Community oriented approach | One Stop Shop Strategy | - Same Sex Marriage Prohibition Act - Criminalisation of homosexuality and same-sex - Peer to peer and support strategy as the basis for service delivery  - Provision of HIV prevention, care, and treatment in a safe place.  - Peer driven linkage and case management strategies |
| International AIDS Society (IAS), 2018 (43) | Decision framework | Decentralizing ART delivery to the community through: | -Provision of client centered care - Drop in center - CBO-led outreach services - Engaging peers in ART delivery | - Safe space - Provision of culturally sensitive services - Criminalisation policy - High mobility of KP - Sensitization and training on KP needs - Access to justice and legal support - Age of consent to services - Anti-stigma and anti-discrimination policy - Provision of KP friendly services, programme led by KPs, - Meaningful participation - Prevention of violence against KPs Training and sensitization of HCW - ART delivery and psychosocial support in group or by a peer  -Network of peers and network of DSD providers |
|  |  | (client-managed group model and out-of-facility individual model) peer-led outreaches, |  | - High level discrimination - Adolescent and young FSWs  - FSW with disability  - Safe space - Provision of Gender responsive services - KP sensitization trraining for law enforcement officers - KP Community mobilisation - Mgt of gender based violence - Mutually supportive relationship between implementing partners,  - FSWs and service providers - Provision of KP friendly health services - KP community participation and ownership  - Peer education |
| National Agency for the Control of AIDS, 2020 (28) | Guidelines | One Stop Shop Model of Integrated Service Delivery | OSS, support group, Mobile community ARV, Referral clinics, home-based ART) | - Stigma free environment that is conducive, gender responsive, client friendly and safe for key populations to access comprehensive |
| National Agency for the Control of AIDS, 2020 (27) | Guidelines | One Stop Shop Model of Integrated Service Delivery |  | -Mental health problems among MSM  -High levels of sexual stigma,  -Young MSM and MSM living with disability, --Typology of MSM  -Outreach  -Peer education  -Capacity building for HCWs,  -collaboration with KP-led CBO  -Community mobilisation and dialogue  -Geographic location  -Age (younger MSM) |
| National Agency for the Control of AIDS, 2020 (26) | Guidelines | One Stop Shop Model of Integrated Service Delivery |  | - Integrates task shifting  - Decentralization, integration and simplification of care across the HIV care continuum -Drug use is commoner among the ages of 25 and 39 years  -Low self perceived risk of HIV  -Poor health seeking behaviour  - Socioeconomic status,  -Gender status, sexual orientation and behaviour, outreach (physical and virtual)  -Peer education through IPC  - KP community and participation - Engagement with law enforcement officials |
| National Agency for the Control of AIDS, 2021 (30) | Strategic framework | Differentiated ART Service Delivery | Community-based ART models | - Stigmatization and discrimination  - Criminalizing legal environment - High mobility of KP - Ancillary services - Urban or rural location - Unstable context (e.g. conflict, high migration) and epidemic/pandemic scenarios - High or low HIV prevalence areas, border town, high migration areas, conflict regions - Cost of transportation - Overburdened health facilities - Long waiting time - An enabling environment (safe space) that provides the full complement of services required by this group is pertinent. - Cultural norms and practices (Strong kinship and strong family ties, emphasis on chastity) - Young people live in poverty due to unemployment |
| Federal Ministry of Health, Abuja, Nigeria (18) | Guidelines | Differentiated ART Service Delivery: | -OSS for KP and mobile clinics,  - Mobile ART teams (MART)  - Community pharmacy  - Focal service providers  - Peer-led support group meetings  - KPFH facilities | - Prevalence of HIV (high or low) - Hotspots (KP size and location) - Location of service delivery (rural or urban) - Unstable or challenging settings- conflict areas - High migration area and border towns - Distance from service location, transportation costs, and safety concerns - Over-burdened health facilities - Covid 19 Pandemic (competing needs for human and infrastructural resources, lock downs) |
|  |  |  |  |  |
|  |  |  |  |  |
|  |  |  |  |  |
|  |  |  |  |  |
|  |  |  |  |  |
|  |  |  |  |  |
| Society of Family Health, 2012 (32) | Training Guide | One stop shop clinic Community drop-in-centre Peer outreaches |  | - HCW attitude and bahaviour towards KP - Highly stigmatized and criminalized practice in Nigeria - Limited knowledge of KP size and location - Drivers of sex industry include: increasing economic difficulty in the country, rising unemployment, and the high cost of living - MSW and FSWs are usually between late teenage years and forty in age - Sexual orientation and practices - Strong linkage between sex work and drug use as IDUs can exchange sex for drugs or engage in sex work to support their drug use habit |
| Society of Family Health, 2018 (25) | Report | Community-based OSS | -Stand-alone HIV testing points - Moonlight Mobile clinics - Community-based ART specialized clinics (OSS clinic) - Community drop -in centres - KP Friendly Health Facilities - Supports groups | - Long waiting time and rigid hours for service provision in the health facilities  - Ineffective linkage to ART due OOP cost - HCW attitudes towards KP - Stigma and discrimination - Clients residing in hard to reach and distant communities from the ART centres - Lack of understanding of issues affecting KPs - Partnership with key stakeholders (goverment agencies, implementing partners, Civil Society Organizations/Ccommunity Based Organizations |
| FHI 360/LINKAGES, 2016 (23) | Guidelines | Peer Outreach Community drop-in-center | -HIV testing services - ART initiation and refill - Referral to STI, reproductive health, TB, and viral hepatitis PCT | -KP size estimation and mapping - Power-holders and stakeholders engagement - Advocacy and KP sensitization, - KP engagement and empowerment - KP participation in service delivery, hotspots ,  - Training and support forKP members - Staffing of programs,  - Networks of key poulations - Availability of Guidelines, SOPs, policies on stigma reduction - Monitoring and Evaluation |
| FHI 360/LINKAGES, 2017 (24) | Report: LINKAGES approach and lesson learnt | Peer Outreach Community drop-in-center | HIV testing services ART initiation and refill Referral to STI, reproductive health, TB, and viral hepatitis PCT | - Community engagement and empowerment/capacity building,  - Advocacy - Human rights, gender equality, zero tolerance for stigma, discrimination, and violence - Program management and data use - Stigma and discrimination - Harrassment and viloence |
| W Tun et al, 2019(10) | A quasi experimental Prospective cohort study | Community drop-in center with mobile team | - Community-based ART mobile and home-based platform for FSW  - ART services, enrolment Drug pick-ups - Trainings for providers - Peer escort servivces - Enhanced adherence coEunselling | - Sociodemographic: Age, marital status, education, number of living children,  Income from sex work, average monthly income,  - Traveling outside the region to sell sex. - Internalised stigma, - Only Stable clients are eligible - Perception and knowledge about HIV - Indirect costs (transportation costs, travel time to clinics) - Poor quality of care,  - HCW attitude - Younger FSWs (Age) - Collaboration with government agencies and donors - Aligns with the national HIV&AIDS guidelines |
| Kerrigan et al, 2019 (44) | Community randomised trials | Community drop-in-centre | -Community mobilization activities - Venue-based peer education - Condom distribution, and - HIV counseling and testing service navigation  - Social support to promote HIV treatment access and adherence - SMS and reminder messages to promote care engagement and ART adherence among HIV-positive women. | - Wome aged 18 years or older - Community support and savings groups - Community empowerment - Wrap around services such as cervical cancer screening, community health education fairs, workshop with local police and justice sector officials, GBV support - Provider sensitivity trainings - Workshops on topics such as stigma, discrimination, and GBV; family planning; HIV/sexually transmitted infection prevention including condom negotiation; ART adherence; financial security; and sex worker rights and community mobilization strategies  HIV clinical care provider and police sensitivity trainings; |
| Yves Lafort (45) | Mixed methods design | Stand-alone drop-in clinic (the ‘Night Clinic’)   Peer outreaches . | A ‘diagonal’ intervention which combined strengthening of FSW-targeted services (vertical) with making public health facilities more FSW-friendly (horizontal). Peer outreach and some SRH services (information, condoms, STI treatment, family planning and HIV testing) | - Collaboration with NGO and the District Health Department - Capacity building and empowerment for FSW CBOs - Insufficient institutional capacity - Poor buy-in from government and private partners, - Dependency on donors. - Lack of endorsement by national policy makers for the targeted, vertical component. - Sensitizing and training of HCWs as focal persons in health facilities - Geographic location and access (Mining industry and major transport routes) |
| Ramadhani et al, 2018(41) | Prospective cohort study | Community-based health centre for MSM | - | -Collaboration with key stakeholders (Kenyan National AIDS and STD Control Pro-gramme. Research counsellors and clinical officers)  - Peer education and mentoring  - Provide Next Step Counselling approach using motivational interviewing technique |
| Cowan et al, 2018 (34) | Randomised controlled Trial | Community drop-in center for FSW | Drop-in-centres plus  Adherence sisters program (for those on ART & PrEP) Onsite ART & PrEP initiation | -FSW aged 18 years or older - Frequency of visit-monthly |
| Graham et al, 2018 (46) | Randomised controlled Trial | Community-based health centre (NSC & peer support) for GBMSM | - Provision of Next Step Counselling approach using motivational interviewing technique | - MSM , age ≥18 years - Duration on ART care  - Peer education |
| Kayode et al, 2020 (47) | Prospective cohort study | Community- based health centre for MSM |  | - MSM sensitivity training (culturally sensitive service delivery) -Standard training in HIV/STI management MSM>/= 16 years - Varying socioeconomic status (with or without phone)  - Capacity building for health professionals  - Provision of wrap around services- condoms and lubricats, STI Rx  - Community mobilisation  - Sensitization training to meet social, legal and sexual health needs of study participants |
| Diallo et al, 2020 (48) | Prospective cohort study | Community-based centre (research HIV & STI clinic dedicated to FSW) | Provision of ART care by health professionals | -High mobility of sex workers  - KP community engagement  -KP community participation (clients’ tracking)  -Provision of wrap around services (STI Rx, PrEP |
| Olawore et al, 2020 (49) | Retrospective cohort | Community drop-in centre) | Enhanced peer outreach within the LINKAGES’ project | - Member of the KP and not previously engaged with an HIV program - POW are trained by local CBOs and implementing partner - PM are not formally trained, but they are familiarized with EPO process, participants selection, and coupons distributions |
| Ibiloye et al, 2018 (11) | Retrspective cohort study | Community-based health centre, Drop-in-centres & ART mobile/outreach (OSS model) | - ART outreaches to hotspots - | - Capacity buidling for health professionals and KP clients (ART training, KP sensitization training) - KP aged 18 years or older - Socioeconomic status (education, unemployment)  Community mobilisation for HIV testing & counselling,and ART |
| APIN, 2019 (50) | Report | Report for the Baseline Organizational Capacity Assessment of KP-Led and KP Friendly CBOs in Benue State for the project-  ‘Ensuring Comprehensive HIV/AIDS Response and Friendly Community Approach (EnCOMPARS Building Sustainable KP-Led and KP’ | -Institutional and capacity building of KP-CBOs - Engagement of KP-CBO - Mentoring and supportive supervision - Provision of comprehensive HIV services | **Micro:** KP type, lack of knowledge of HIV status, High risk sexual behaviour (low use of condoms, multiple sexual partners) **Meso:** Difficulty reaching MSM and PWID, inadequate number of KP-led and KP friendly CBOs, sub-optimal KP community involvement and program ownership,  **CBO context:** governance structure, (experience implementing KP program, knowledge and skills of staff, familiarity with Benue terrain and community members, M&E, gender issues, service delivery, resource mobilisation, HRM, lack of knowledge of KP size **Macro**: |
| Society for Family Health, 2019 (51) | RFP | Comprehensive community-based HIV Service Package | -OSS  -Public/private health facilities  - Peer-led outreach | -Empowerment-based, peer-led outreach with behavioral components that build long term self-efficacy  - - Provision of condoms and lubricant including increasing access through other market approaches  - Identification and capacity building of KP-Led community-based organizations  - Working with KP secretariat to effectively deliver on all the objectives.  - Provision of enabling legal environment for Key Population Programming through promotion of human right, access to justice, legal aid services and reduction of stigma and discrimination. |
| USAID, Nigeria (52) | NOFO | OSS Model (including drop in centre) | OSS Model + community mobilisation+wrap around services (mental health, STI Rx, violence mitigation) | - Sexual identity  - Age, education, non use of condom, high HIV prevalnce among KP, PrEP use  -Stigma and discrimination  - Inaccurate size estimation  -Non-disclosure of HIV statu  - Use of virtual technology to attract clients |
