# Supplementary material for: Initial programme theory for community-based ART delivery for key populations in Benue State, Nigeria: a realist evaluation study
Source: BMC Public Health. 2023 May 12;23:870. doi: 10.1186/s12889-023-15774-w (PMC10176666; doi:10.1186/s12889-023-15774-w)
Supplement: Supplementary file 4 — Additional file 4: Table 3. Topic guide for in-depth interview with programme designers/managersin the community-based HIV programme for key populations in Benue State,Nigeria. [file 12889_2023_15774_MOESM4_ESM.docx]

**Supplementary Table 3. Topic guide for in-depth interview with programme designers/managers in the community-based HIV programme for key populations in Benue State, Nigeria**

| **Elements** | **Actions/Questions** | **Codes** |
| --- | --- | --- |
| Arrival | Settling in of study participants |  |
| Introduction | Introduction of interviewer/ interviewees  Objectives setting  Request for permission to audio-record and to take notes  Discuss the information leaflet and consent form  Obtained informed consent  Questions for clarification |  |
| Equipment Check | Check the tape recording and ensure it is functional |  |
| Ice-breaker | Ask any context-specific funny question to relax the interviewee  Check whether they have time or whether to reschedule? |  |
| About the respondents | Q: How long have you been supporting the program and in what capacity?  Q: What are your responsibilities in the community-based HIV programme for key populations (MSM, FSW, PWID, and TG) ? |  |
| Description of the intervention/strategies and activities | Q: In which year was the community-based HIV programme for KP established?  Q: What is the history behind the establishment of the community-based HIV programme for KP?  *Probe for what they know given their time with the programme?*  *Probe for who knows more, who was there since the beginning?*  Q: From a manager’s perspective, what was the purpose of establishing community-based HIV programme for key populations ?  Q: What were the successful strategies of the programme?  *Probe for what did not work or worked less and why?*  Q: What are the activities provided by the community-based HIV programme  *Probe for ART initiation, refill, peer education viral load)?*  Q: What type of ART service delivery models are provided by the community-based HIV programme  *Probe for mobile ART outreaches, drop in centre, and OSS clinic?* |  |
| Actors | Q: What category of staff are recruited to work in this program and what are their roles?   - 1. Did staff receive any specific type of training?   2. Were their staff from health facilities in the vicinity that switched jobs – because they heard positive things about the programme?   3. What was their reaction on their first day at work?   4. Was it difficult for them initially to work with HIV patients as lay workers or counsellors? |  |
| Programme outcomes | Q: What is the most important single achievement of the community-based HIV programme for KP?  Q: From a manager’s perspective, how do you see the community-based HIV programme benefitting individual patients in the programme ?   - 1. *Can you give two examples of benefits to individual patients?*   Q: From a managers perspective, can you comment about the role of community-based HIV programme in improving HIV epidemic (new HIV infection and mortality rate) at the facility and community level ?  Q: How do you see the community-based HIV programme benefitting the local community and the health systems?   - 1. *Can you give two examples of benefits to the community and the health system ?*   Q: In your opinion, how do you think provision of ART in the community setting for KP will improve medication adherence and retention in HIV care ?   - 1. *Probe for a positive group atmosphere, solidarity between KP that emerged and improved adherence* |  |
| Mechanisms | Q: What resources do you think the program offers to key populations that bring about a change in outcome and behaviour ?  *Probe for friendship, trust, solidarity, feeling of not being alone*  Q: What was the initial reaction from the communities when the programme was introduced?  *Probe for:*   - 1. *KP community reaction*   2. *What did community leaders say?*   3. *What about the local authorities? The police? Other influential people, the school teachers, and etc..*   Q: Did this programme contribute to solidarity (providing support to each other) within the KP subgroup and between different KPs subgroups? |  |
| Contextual factors | Q: From your perspective as a manager, what are the main barriers to the community-based HIV programme for key populations?  Q: From a managers perspective, what are some of the factors that facilitate the achievement of the community-based HIV programme for KP in improving health outcomes?  Q: What are the socio-political situation affecting the implementation of the community-based HIV programme for KP?  Q: Are there other issues that affect the implementation of the programme?  *Probe for Police harassment, stigma / discrimination religious communities/leaders*  Q: Were there issues that the local community would see as disadvantages (may be more sex work in certain neighbourhoods).  Q: What assistance would this programme need from the national or state level in order to succeed? What can be done to support this programme (more than now) |  |
| Conclusion | Q: Would you like to add anything else?  Q: Do you have a question for me?  Q: Any things you would still like to add? |  |
