# Supplementary material for: Initial programme theory for community-based ART delivery for key populations in Benue State, Nigeria: a realist evaluation study
Source: BMC Public Health. 2023 May 12;23:870. doi: 10.1186/s12889-023-15774-w (PMC10176666; doi:10.1186/s12889-023-15774-w)
Supplement: Supplementary file 5 — Additional file 5: Table 4a. Intervention and Actors. Table 4b. Outcomes. [file 12889_2023_15774_MOESM5_ESM.docx]

**Supplementary Table 4 (4a & 4b)**

**Table 4a. Intervention and Actors**

| **CBART Models** | **Service provided** | **Actors** |
| --- | --- | --- |
| - One Stop Shop Clinic - Drop-in-center and venue-based approaches - Mobile ART Teams - Mobile Clinics - Peer-led support group meetings - Community Pharmacy - Focal Service Providers | - Provision of community-led HIV services - Peer driven intervention/peer driven service providers - Provision of ART and treatment in a safe place - Peer to peer support strategy - ART delivery and psychosocial support in group or by a peer - Ancillary services (wrap around services) - KP sensitization training | - KP clients (FSW, MSM, PWID) - Clinically stable and unstable KP clients - Trained health care providers (clinician, nurses, lab scientist, clinical psychologist) - KP Community/Network - PLHIV association - Peer educators, Case Managers - Community health workers - Government agencies (National Agency for the Control of AIDS, NASCP) - Lay HCW, - Government agency (NACA and NASCP) - Service Delivery Point managers |

**Table 4b. Outcomes**

| **Immediate** | **Intermediate** | **Long term** |
| --- | --- | --- |
| - Minimise stigma and discrimination - Short waiting time - Less travel expenses - Early access to treatment - Increased accessibility and acceptability of services for key populations - Change in behaviour and attitude - Utilisation of HIV services - Higher service quality satisfaction compare to public health facility - Reduced burden on health facilities | - Improved engagement in care - High uptake of HIV testing and linkage to ART - Medication adherence - Retention in care - Viral load suppression | - Improved access, quality of care and efficiency of health systems - Reduced new HIV infection, mortality, and morbidity |
