# Supplementary material for: Initial programme theory for community-based ART delivery for key populations in Benue State, Nigeria: a realist evaluation study
Source: BMC Public Health. 2023 May 12;23:870. doi: 10.1186/s12889-023-15774-w (PMC10176666; doi:10.1186/s12889-023-15774-w)
Supplement: Supplementary file 6 — Additional file 6: Table 5. Classification of Mechanisms. [file 12889_2023_15774_MOESM6_ESM.docx]

**Supplementary Table 5. Classification of Mechanisms**

| **KP Clients** | **Health care providers** | **SD (organisational)** | **Systems** |
| --- | --- | --- | --- |
| - Mutually supportive relationship between Implementing partners, KP (i.e. FSWs) and service providers (bonding) - KP community participation and ownership - Feeling safe and secured - Bonding, solidarity among KP - Recognition lay knowledge (KP themselves recognized as lay workers / peer counsellors), increasing self-worth, confidence - Feeling of importance and relevance (sense of community belonging) - Trust in the system (privacy and confidentiality) - Positive group dynamics - Buy-in - Meaningful participation - Encouraged/gentle push (through peer support) - Motivation | - Buy-in - Recognition by KP -motivation-meaningful work - Better social interaction with KP - Mutual recognition KP-HWC | - Provision of KP friendly health services (friendliness towards KP) - Stigma free environment that is conducive, gender responsive, client friendly and safe for key populations to access comprehensive | - Buy in (policy makers) - Meaningful participation and involvement in KP programmming - Above-site mentoring and supervision |
