# Supplementary material for: Initial programme theory for community-based ART delivery for key populations in Benue State, Nigeria: a realist evaluation study
Source: BMC Public Health. 2023 May 12;23:870. doi: 10.1186/s12889-023-15774-w (PMC10176666; doi:10.1186/s12889-023-15774-w)
Supplement: Supplementary file 7 — Additional file 7: Table 6.Contexts. [file 12889_2023_15774_MOESM7_ESM.docx]

**Supplementary Table 6. Contexts**

| **Micro** | **Meso (as organisational -at SD point level)** | **Macro (wider societal and local context- embeddedness in local community)** |
| --- | --- | --- |
| - Age of consent to HIV service delivery - Adolescent and young FSWs,  FSW with disability Individual level stigma and discrimination - High mobility of KP - High cost of transportation - Travel distance - Cost of Service - Fear of disclosure of HIV status (in public health - Place of residence (rural, urban, border town) | **Package of Care (Comprehensive HIV care & treatment services) through One stop shop**   - HIV Testing Services, antiretroviral therapy, Management of opportunistic infections including STI   **Outreach and Mobile ART services**   - Drop-in services and weekend and/ or night service times. - Outreach including venue-based and home-visiting servic**e**   **KP friendly health services:**   - Provision of community-led HIV services - Peer driven intervention/peer driven service providers - Provision of ART and treatment in a safe place - Peer to peer support strategy - ART delivery and psychosocial support in group or by a peer   **Stigma and discrimination in the healthcare facility and community**   - HCW attitude (unfriendly and stigmatizing HCW) - Homophobic environment - Social networking/capital (Network of peers and Network of DSD providers)   **Others**   - Ancillary services (wrap around services) - Sensitization and training of HCWs and law enforcement agencies on KP needs - Community mobilisation | - High or low HIV prevalence areas - Unstable context (e.g. conflict region, high migration areas) and epidemic/pandemic scenarios - Absence of protective legislation - Criminalisation policy - Politics (politicians supporting / resisting programme) - Community associations, religious associations, community leaders, preachers - Harassment from Police and other security forces,… |
